# The expression profile and prognostic significance of eukaryotic translation elongation factors in different cancers

**DOI:** 10.1371/journal.pone.0191377

**Published:** 2018-01-17

**Authors:** Md. Khurshidul Hassan, Dinesh Kumar, Monali Naik, Manjusha Dixit

**Affiliations:** School of Biological Sciences, National Institute of Science Education and Research, HBNI, Bhimpur- Padanpur, Jatni, Khurda, Odisha, India; University of South Alabama Mitchell Cancer Institute, UNITED STATES

## Abstract

Eukaryotic translation factors, especially initiation factors have garnered much attention with regards to their role in the onset and progression of different cancers. However, the expression levels and prognostic significance of translation elongation factors remain poorly explored in different cancers. In this study, we have investigated the mRNA transcript levels of seven translation elongation factors in different cancer types using Oncomine and TCGA databases. Furthermore, we have identified the prognostic significance of these factors using Kaplan-Meier Plotter and SurvExpress databases. We observed altered expression levels of all the elongation factors in different cancers. Higher expression of EEF1A2, EEF1B2, EEF1G, EEF1D, EEF1E1 and EEF2 was observed in most of the cancer types, whereas reverse trend was observed for EEF1A1. Overexpression of many factors predicted poor prognosis in breast (EEF1D, EEF1E1, EEF2) and lung cancer (EEF1A2, EEF1B2, EEF1G, EEF1E1). However, we didn’t see any common correlation of expression levels of elongation factors with survival outcomes across cancer types. Cancer subtype stratification showed association of survival outcomes and expression levels of elongation factors in specific sub-types of breast, lung and gastric cancer. Most interestingly, we observed a reciprocal relationship between the expression levels of the two EEF1A isoforms viz. EEF1A1 and EEF1A2, in most of the cancer types. Our results suggest that translation elongation factors can have a role in tumorigenesis and affect survival in cancer specific manner. Elongation factors have potential to serve as biomarkers and therapeutic drug targets, yet further study is required. Reciprocal relationship of differential expression between EEF1A isoforms observed in multiple cancer types indicates opposing roles in cancer and needs further investigation.

## Introduction

Cancer is a multifactorial disease wherein the expression levels of many genes, particularly of those regulating growth and division of cells, are altered [1]. The expression and functionality of these genes is regulated at multiple levels viz. transcription, RNA polyadenylation and splicing, mRNA export from the nucleus to the cytoplasm, mRNA degradation, translation of mRNA to protein and post-translational modifications. In the recent years, much attention has been given to unravel the regulation of gene expression at the level of translation, with regards to controlling the process of carcinogenesis [1]. The process of translation is a complex one, wherein dozens of different translation factors are involved, each regulating the specific steps of initiation, elongation and termination [2, 3]. Although all the steps involved in translation are tightly regulated, yet the rate limiting step is that of initiation and hence has garnered the most attention in targeting cancer [4]. On the other hand, limited studies have investigated the contribution of translation elongation factors in the onset and progression of different cancers. In the recent years, a translation elongation factor of the eukaryotic elongation factor 1 alpha (EEF1A) family, namely eukaryotic elongation factor 1 alpha 2 (EEF1A2) has attracted much attention due to multiple studies highlighting its role as an oncogene, across different cancers [5–10]. The other EEF1A isoform viz. EEF1A1 has been reported to play a pro-apoptotic function in certain reports [11–14] while anti-apoptotic in others [15–18]. However, it has been assigned a minor role in the onset of cancer, opposed to the other EEF1A isoform viz. EEF1A2 [7]. Eukaryotic translation elongation factor 1 beta 2 (EEF1B2) has been reported to be downregulated in IR-induced senescence in MCF7 cells [19]. Eukaryotic translation elongation factor 1 gamma (EEF1G) has been reported to be overexpressed in gastric carcinoma [20], colon adenocarcinoma [21], and pancreatic cancer [22]. Eukaryotic translation elongation factor 1 delta (EEF1D) has been linked with mediating epithelial mesenchymal transition (EMT) in oral squamous cell carcinoma [23] and its overexpression is associated with worse overall survival and progression free survival in medulloblastoma [24]. Eukaryotic translation elongation factor 1 epsilon 1 (EEF1E1) has been identified as a putative tumor suppressor due to its downregulation in gastric and colorectal cancer [25]. Eukaryotic translation elongation factor 2 (EEF2) was found to be overexpressed at the protein level, in gastrointestinal cancers [26] and in hepatocellular carcinoma (HCC) [27]. Higher serum EEF2 levels were observed in non-small cell lung cancer patients [28]. EEF2 was also identified as a tumor associated antigen overexpressed in the majority of several types of cancers and plays an oncogenic role in cancer cell growth [29]. These preliminary studies suggest that in addition to EEF1A2, other translation elongation factors are involved in multiple human cancers.

However, apart from EEF1A2, the prognostic significance of other translation elongation factors remain largely unexplored across different cancer types. EEF1A2 expression is reported to be associated with good prognosis in breast cancer [30] and non-small cell lung cancer [31] On the contrary, it predicts worse survival in pancreatic [32], ovarian [33] and gastric cancers [34]. Therefore, a comprehensive analysis of association of expression levels of all translation elongation factors across different cancers is essential to ascertain their relevance as potential therapeutic targets or prognostic biomarkers.

In summary, studies so far indicate contradictory differential expression of translation elongation factors in different cancers, some factors are not at all studied in any cancer, some factors are studied in one or two cancers; moreover, there is no study on the prognostic significance of most of the factors. This study will first time unravel the common pattern of differential expression of translation elongation factors across cancers and, provide prognostic value and it’s common trend of all elongation factors in cancers. Keeping these lacunae in hindsight, we have undertaken a pan-cancer approach for comprehensive analysis of expression levels of seven elongation factors namely, EEF1A1, EEF1A2, EEF1B2, EEF1G, EEF1D, EEF1E1 and EEF2, using publicly available databases (Oncomine and TCGA). Additionally, we have determined the prognostic significance of these factors in different cancer types by making use of survival data available in Kaplan-Meier Plotter (KM Plotter) and SurvExpress databases.

## Material and methods

### Oncomine analysis

To analyse the transcript level of the genes of interest in different cancer subtypes, Oncomine [https://www.Oncomine.org, Compendia biosciences, Ann Arbor, MI, USA] i.e. an online microarray database, was used [35]. The thresholds selected for inclusion of studies from the database were as follows—p value ≤ 0.01, fold change ≥ 2 fold and gene rank ≤ 10%. For each individual gene, comparison of expression levels was performed by carrying out cancer vs. normal analysis.

### TCGA analysis using UCSC Xena browser

Another database that was used for analysing the transcript levels of genes of interest was The Cancer Genome Atlas (TCGA) i.e. a collection of web-based tools that visualize, integrate and analyse cancer genomics and its associated clinical data. Integrin mRNA HiSeq expression data for lung, breast, colorectal, gastric, brain, prostate, and liver were downloaded using the latest UCSC Xena browser (http://xena.ucsc.edu/) version: 2016-08-16. Student’s t-test was performed to ascertain the differences in mRNA levels of genes of interest, between tumor and normal tissues. Subsequently, Box-plots were made using graph-pad prism software.

### Kaplan-Meier plotter analysis

We used Kaplan-Meier (KM) Plotter (http://kmplot.com/analysis/), a database that integrates gene expression data and clinical data [36], to obtain survival data for gastric, lung, breast and ovarian cancer, in relation to expression levels of genes of interest. Kaplan Meier plotter has information of 54,675 genes on survival, including 5143 breast, 1816 ovarian, 2437 lung and 1065 gastric cancer patients with a mean follow-up of 69 / 40 / 49 / 33 months, respectively. Briefly, the best specific probes (JetSet probes) for each gene of interest were individually entered to obtain KM plots. Information on overall patient survival (OS), first progression (FP), post progression survival (PPS), distance metastasis free survival (DMFS) and relapse free survival (RFS) was extracted. Furthermore, information on number of cases along with median values of mRNA expression levels, hazard ratios (HR) with 95% confidence intervals (CI) and p-values were extracted from the KM plotter webpage and considered significant having p-values ≤ 0.05.

### SurvExpress database analysis

SurvExpress database [37] (http://bioinformatica.mty.itesm.mx:8080/Biomatec/SurvivaX.jsp) was used for obtaining survival data for seven other cancers (brain and CNS, prostate, colorectal, pancreatic, kidney, head and neck, liver), for which information was not available on KM plotter database. TCGA datasets were considered for analysis because of the presence of both desirable probes and larger sample size (> 200 patient). The hazard odds ratio with 95% confidence interval (CI) having p-values ≤ 0.05 was considered significant.

### *In silico* analysis of promoters for transcription factor binding sites

Promoter sequences of the translation elongation factor genes were manually obtained from the Eukaryotic Promoter Database (EPD) [38] These sequences were analysed with PROMO v3 (http://alggen.lsi.upc.es/cgi-bin/promo_v3/promo/promoinit.cgi?dirDB=TF_8.3). PROMO is an online tool for identification of putative transcription factor binding sites (TFBS) in DNA sequences [39] [PMID: 11847087], it uses version 8.3 of TRANSFAC. Graphical representation of the common transcription factors binding sites, present in the input sequences with a dissimilarity margin less or equal to 15%, was obtained.

## Results

**Fig 1:**
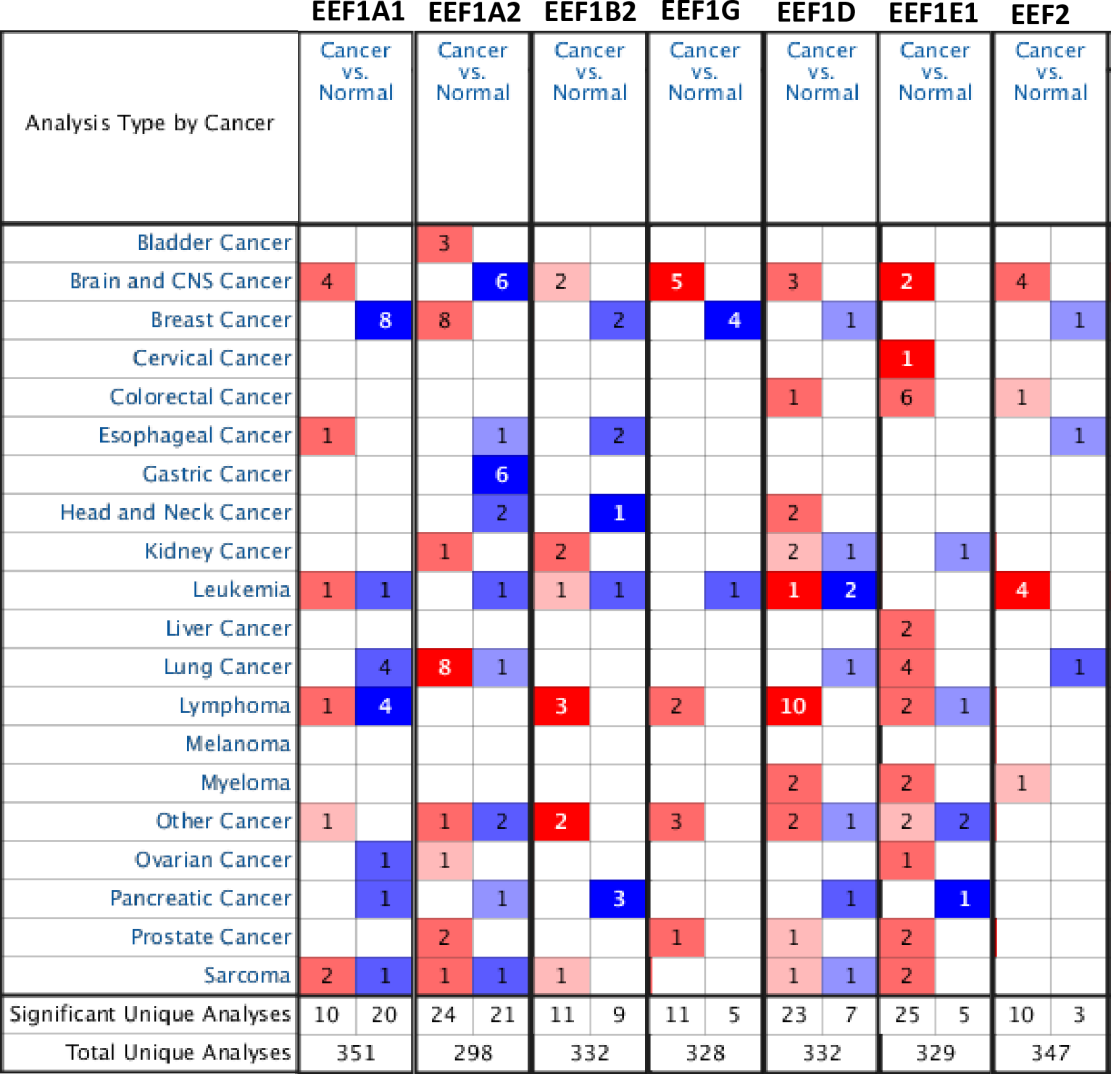
Oncomine analysis of expression levels of elongation factors across different cancers. The differences in expression levels of the genes between tumor and normal tissue are summarised in the figure. The number of unique analyses satisfying the thresholds (p-value ≤ 0.01; fold change ≥ 2; gene rank ≤ 10%; data type: mRNA) are indicated in the colored cells. Red cells represent overexpression of the target gene, in tumor tissues compared to normal, whereas blue cells indicate downregulation of the same. Gene rank is depicted by the color depth in the cells.

Initially, we performed Oncomine analysis to investigate differences in the mRNA levels of each elongation factor, between tumor and normal tissues in various cancers. A quick glance at the result of this analysis in Fig 1 shows that there are a total of 351, 298, 332, 328, 332, 329, 362 and 347 unique analyses for EEF1A1, EEF1A2, EEF1B2, EEF1G, EEF1D, EEF1E1 and EEF2, respectively. For EEF1A1 expression, a total of 30 studies showed statistically significant difference between tumor and normal samples, across all cancer types. In most of the datasets, EEF1A1 mRNA levels were significantly decreased in tumor as opposed to normal tissues. The most notable among these are breast, lymphoma and lung cancer wherein EEF1A1 mRNA levels were significantly reduced in tumor cases in a total of eight, four and four unique analyses, respectively. On the contrary, the other EEF1A isoform namely EEF1A2, displayed significantly elevated mRNA levels in tumor tissues in 24 datasets and the opposite trend in 21 datasets. For EEF1B2, 11 datasets showed increased expression in tumor tissues, whereas nine datasets showed significantly reduced levels in tumors, compared to control tissue. Likewise, increased levels of EEF1D transcript were observed in majority of datasets (23) across multiple cancer types, however reduced levels were observed in seven datasets. In tumor tissue, overexpression of EEF1E1 was found in 25 datasets, whereas downregulation was observed only in five sets. Similarly, EEF2 was expressed at higher levels in 10 datasets, conversely, reduced levels were observed in three unique analyses. As we observed significant changes in the expression of all elongation factors, further we looked for differential expression in specific cancer types and their subtypes.

### Expression levels and prognostic significance of elongation factors in breast cancer

As revealed by comprehensive transcriptional studies in the recent years, breast cancer has multiple intrinsic subtypes viz. luminal A, luminal B, HER2-enriched and basal-like; each with distinct morphologies and clinical implications. Keeping in view the importance of this subtype stratification with regards to prediction of treatment sensitivity and survival outcome, we have shown data for subtype analyses wherever possible.

EEF1A1 was found to be significantly downregulated in ductal breast carcinoma, compared to normal tissues, in Radyanvi and Richardson datasets. Transcript levels of EEF1A1 were also significantly reduced in lobular breast carcinoma, in Zhao dataset. Further, multiple datasets including Karnoub, Ma, Zhao and Finak, indicated that EEF1A1 was significantly downregulated in invasive breast carcinomas. On the contrary, EEF1A2 mRNA levels were significantly elevated in one analysis of Radyanwi dataset and five analyses of Curtis dataset across different subtypes, including invasive mixed breast carcinoma, mucinous breast carcinoma, invasive ductal breast carcinoma, invasive ductal and invasive lobular breast carcinoma, invasive lobular breast carcinoma and tubular breast carcinoma. For EEF1B2, two datasets viz. Ma Breast and Finak Breast, showed reduced levels of EEF1B2 in invasive ductal breast carcinoma and invasive breast carcinoma respectively, compared to the normal. Likewise for EEF1G expression, all the four datasets showed a significant downregulation in the tumor subtypes, including invasive ductal breast carcinoma and ductal breast carcinoma *in situ*. No dataset showed significant difference in expression, between tumor and normal group, for EEF1E1. In a single analysis of Finak’s dataset, both EEF1D and EEF2 mRNA levels were significantly reduced in invasive breast carcinoma, compared to normal tissue. All the results with corresponding p-values for statistical significance are summarized in S1 Table.

**Fig 2:**
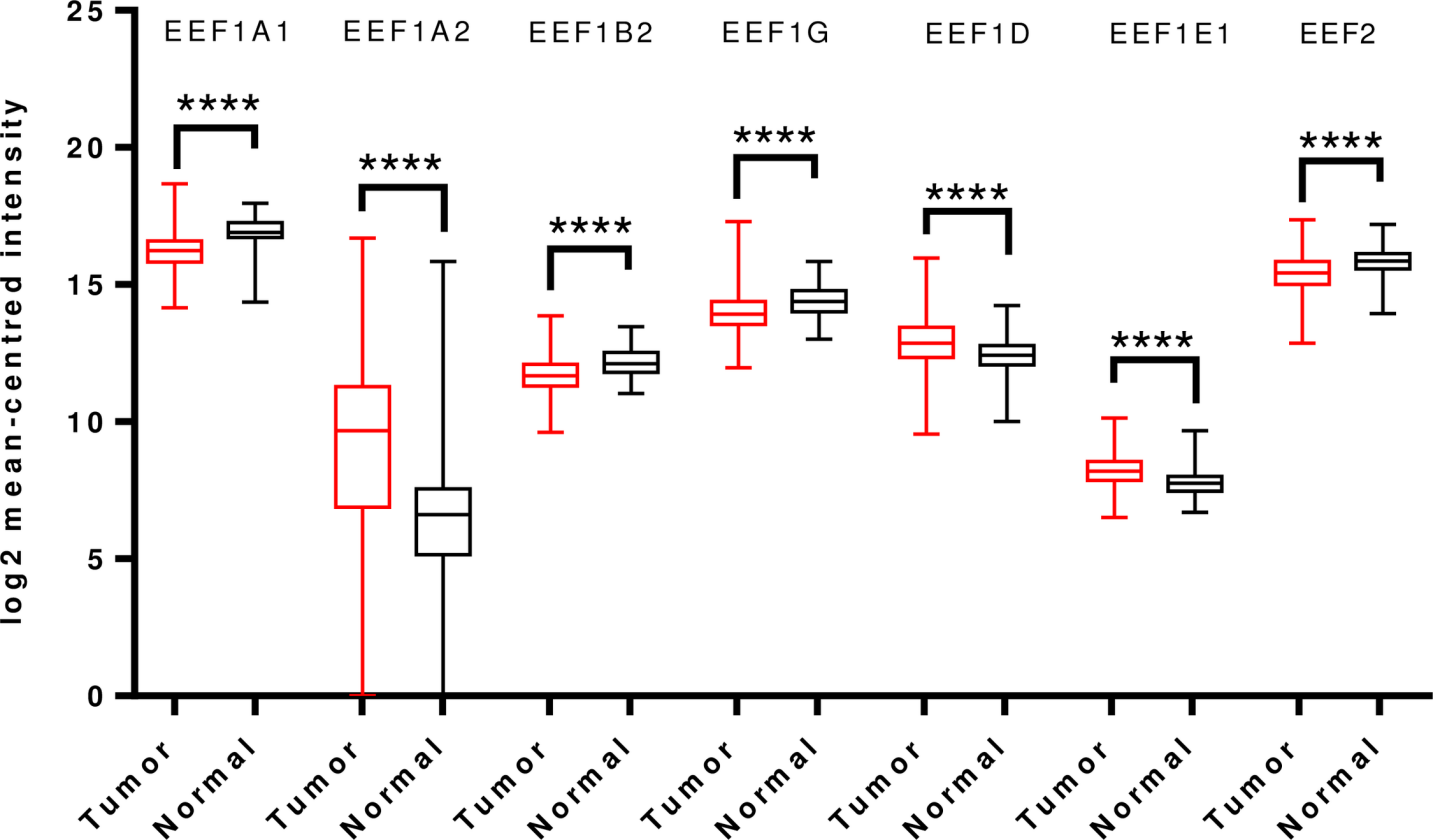
Analysis of mRNA expression levels of elongation factors in breast cancer using TCGA mRNA HiSeq expression data. Box-whisker plots represent the differences in transcript levels of each elongation factor, between normal and tumor samples. The median value is represented by the middle line in the boxes. Statistical differences were ascertained by two-tailed Student’s *t*-test using graph-pad prism software. ****p ≤ 0.0001.

To further validate and cross-examine the observations made in Oncomine database, the mRNA HiSeq expression data from TCGA dataset for breast cancer, containing 1097 tumor cases and 114 normal cases, was examined for differential mRNA levels of the elongation factors in tumor tissue. As shown in Fig 2, transcript levels of EEF1A2 and EEF1D were significantly overexpressed and those of EEF1A1, EEF1B2, EEF1G, EEF1E1 and EEF2 were downregulated in the same. This finding is mostly in agreement with the Oncomine analysis, except for EEF1D wherein reverse trend was seen (Fig 2).

#### Prognostic significance

We assessed the prognostic significance of these factors in breast cancer using the KM plotter dataset (www.kmplot.com). The chosen probe IDs for each gene have been listed in S2 Table. The analysis revealed that higher expression of EEF1A1 was significantly associated with relapse free survival (RFS) and distant metastasis free survival (DMFS), but not with overall survival (OS) or post progression survival (PPS), in breast cancer. EEF1A2 expression was not found to be significantly correlated with OS, PPS, RFS or DMFS in breast cancer patients. However, higher expression of EEF1A2 showed a weak correlation, although not statistically significant (p = 0.07), with worse DMFS. Elevated levels of EEF1B2 mRNA predicted better OS and DMFS. Likewise, increased EEF1G transcript levels showed positive correlation with DMFS and RFS in breast cancer. The higher EEF1D expression on the other hand, predicted worse RFS in breast cancer patients. Higher EEF1E1 expression showed significant correlation with RFS and DMFS but not with either OS or PPS. A very significant correlation with better OS, RFS and DMFS was observed in case of higher EEF2 expression in patients.

Further analyses were carried out based on the four intrinsic subtypes, described previously. Interestingly, upregulated EEF1A1 levels were significantly associated with worse OS and PPS in basal subtype. Higher EEF1A2 levels were significantly associated with worse DMFS in luminal A type but favourable PPS in Her2+ subtype. Higher EEF1B2 expression predicted worse DMFS in basal type, but better prognosis in luminal A (OS, DMFS) and luminal B (OS) subtypes. Elevated EEF1G levels correlated significantly with good prognosis in luminal A subtype. On the other hand, upregulation of EEF1D transcripts predicted poor OS and RFS in basal subtype and, poor RFS in luminal A subtype. Higher EEF1E1 levels were strongly associated with poor RFS in both the luminal subtypes. Higher levels of EEF2 were significantly associated with poor RFS and PPS in basal subtype, but better RFS and DMFS in luminal subtypes. The results of the survival analysis in breast cancer subtypes have been summarised in S3 Table.

### Expression levels and prognostic significance of elongation factors in lung cancer

Lung cancer can be broadly divided into two major subtypes, namely small cell lung cancer (SCLC) and non-small cell lung cancer (NSCLC). NSCLC can be further subdivided into adenocarcinoma, squamous cell carcinoma, and large cell carcinoma. Another less frequent (< 5% of all lung cancer cases) type of lung cancer subtype is the lung carcinoid tumor, which are also called as neuroendocrine tumors.

Further analysis was carried out in datasets available in Oncomine, based on aforementioned subgroups. In parallel to trend seen in breast cancer, EEF1A1 mRNA levels were significantly down regulated in tumor tissue, in two studies (Hou and Garber). Analysis in subtypes showed that EEF1A1 expression was reduced in lung adenocarcinoma, small cell carcinoma (both in Garber’s dataset) and squamous cell lung carcinoma (Hou’s dataset).

On the other hand, a sizeable number of datasets (eight in total) spanning across five studies (Bhattacharjee, Steerman, Beer, Su, Selamat) indicated increased transcript levels of EEF1A2 in lung cancer. In all the five studies, EEF1A2 was elevated significantly in lung adenocarcinoma subtype. Furthermore, EEF1A2 was significantly upregulated in lung carcinoid tumor, small cell lung carcinoma and squamous cell lung carcinoma subtypes, in Bhattacharjee’s dataset.

EEF1B2, EEF1G and EEF2 mRNA levels did not show any significant difference between tumor and normal groups, in any of the datasets. On the other hand, EEF1D transcript levels were reduced in lung carcinoid tumor in Bhattacharjee’s dataset. Like EEF1A2, EEF1E1 transcript levels were also consistently elevated in small cell lung carcinoma, lung carcinoid tumor and squamous cell lung carcinoma subtypes as per Bhattacharjee’s study and, in large cell lung carcinoma, according to Hou’s dataset. All the results with corresponding p-values for statistical significance are summarized in S4 Table.

**Fig 3:**
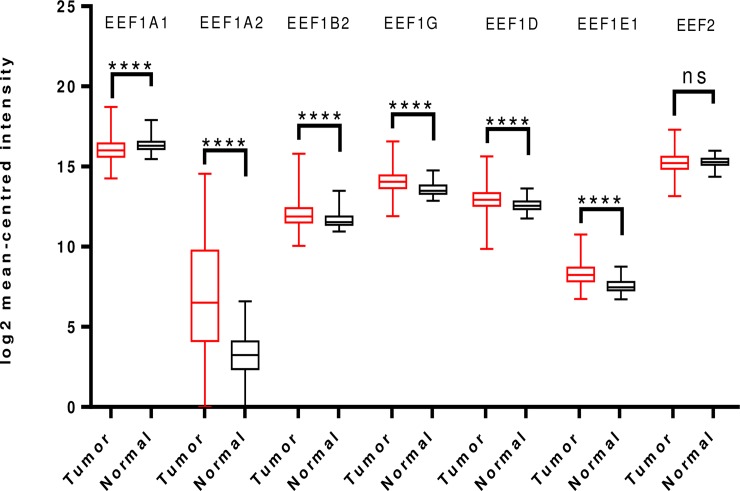
Analysis of mRNA expression levels of elongation factors in lung cancer using TCGA mRNA HiSeq expression data. Box-whisker plots show the differences in transcript levels of each elongation factor, between normal and tumor samples. The median value is represented by the middle line in the boxes. Statistical differences were ascertained by two-tailed Student’s t-test using graph-pad prism software. ****p ≤ 0.0001, ns–non significant.

Examination of TCGA dataset revealed overexpression of all the translation elongation factors being studied, except EEF1A1 which was downregulated and EEF2, which didn’t show any significant difference in expression between the tumor and normal groups (Fig 3).

#### Prognostic significance

To ascertain the prognostic value of these factors in lung cancer, we analysed OS, FP and PPS for each elongation factor (S5 Table). Similar to breast cancer, higher EEF1A1 expression predicted better FP and OS in lung cancer as well. On the contrary, overexpression of EEF1A2 was correlated with worse FP and OS. Worse OS was also observed in patients with higher EEF1B2 expression. Likewise, increased transcript levels of EEF1G and EEF1E1 led to poor OS and FP in lung cancer. EEF1D and EEF2 didn’t show any correlation with any of the survival parameters.

Further analysis in histological subtypes and tumor stages revealed more significant correlations between expression levels and survival outcomes in adenocarcinoma subtype, than in squamous cell carcinoma. Higher EEF1A1 levels in adenocarcinoma correlated with better OS and FP, while higher EEF1A2 levels predicted worse OS and FP. Likewise, elevated mRNA levels of EEF1B2, EEF1G and EEF2 were significantly correlated with worse survival outcome in adenocarcinoma (S6 Table). Survival analysis in stage wise stratified cases revealed that expression values of the elongation factors were more significantly associated with survival outcomes in patients with tumor stage I rather than stage II in almost all the cases.

### Expression levels and prognostic significance of elongation factors in gastric cancer

Gastric cancer mainly comprises adenocarcinoma subtype. The Lauren classification stratified gastric adenocarcinoma into two subtypes i.e. intestinal type and diffuse type. While the former consists of well differentiated tumor cells, tumor cells in the latter type are poorly differentiated and behave aggressively. Mixed type of adenocarcinoma consisting of both intestinal and diffuse type of tumor cell populations are also observed. Human epidermal growth factor receptor 2 (HER2) overexpression is now established as a frequent molecular abnormality associated with gastric and gastroesophageal cancer.

**Fig 4:**
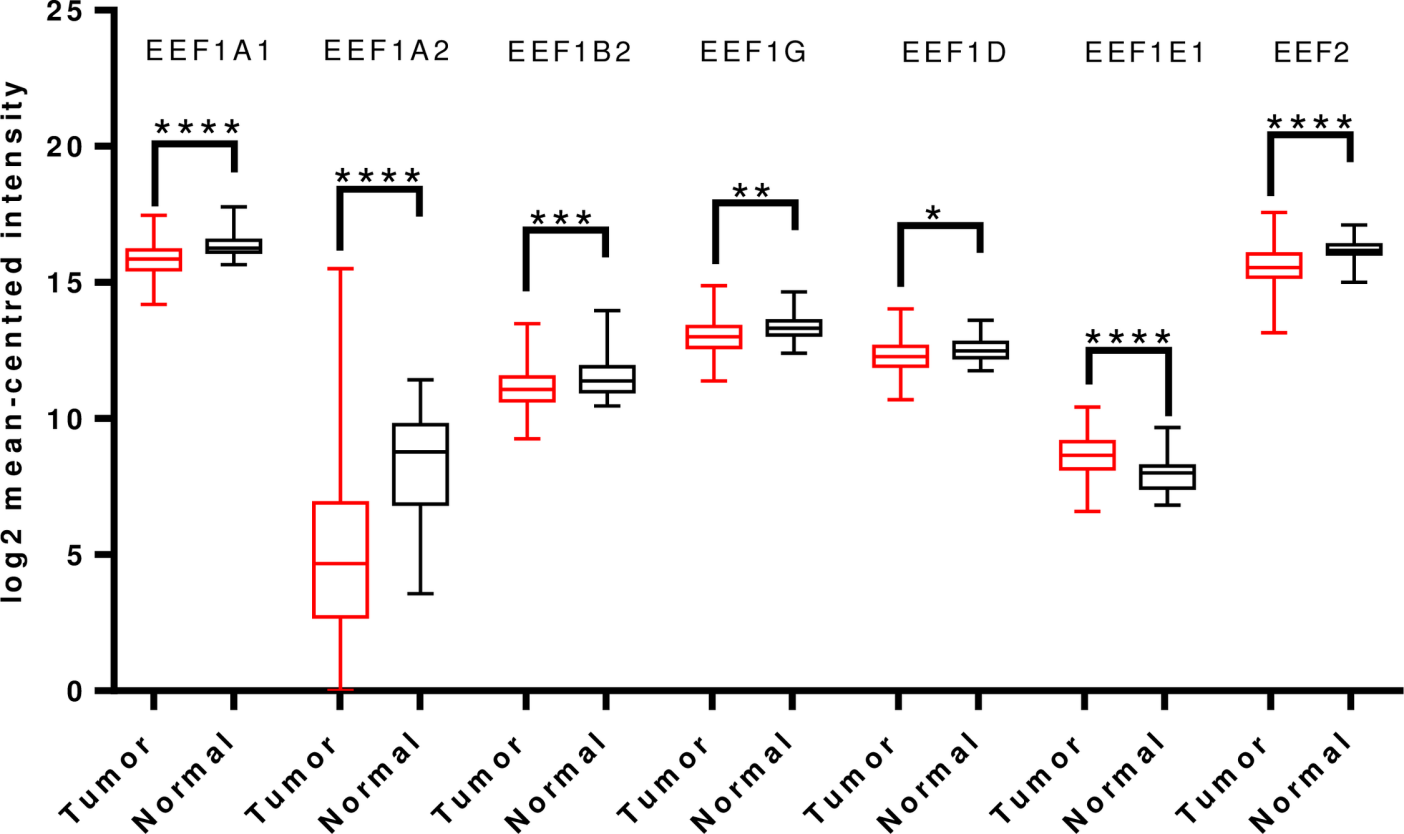
Analysis of mRNA expression levels of elongation factors in gastric cancer using TCGA mRNA HiSeq expression data. Box-whisker plots illustrate the differences in transcript levels of each elongation factor, between normal and tumor samples. The median value is represented by the middle line in the boxes. Statistical differences were ascertained by two-tailed Student’s t-test using graph-pad prism software. ****p ≤ 0.0001, ***p ≤ 0.001, ** p ≤ 0.01, *p ≤ 0.05.

For gastric cancer, apart from EEF1A2 no other translation elongation factor showed significant change in expression in tumors, in Oncomine analysis. In contrast to a previous study reporting overexpression of EEF1A2 in gastric cancer [34], we observed that EEF1A2 mRNA levels were significantly downregulated in a total of four analyses in Cho’s dataset and two analyses in D’Errico’s dataset, spanning across intestinal type, diffuse type and mixed type adenocarcinoma (S7 Table). Overall TCGA analysis confirmed the same, indicating reduced EEF1A2 transcript levels in tumor tissues. mRNA levels of EEF1A1, EEF1B2, EEF1G, EEF1D, EEF2 were also significantly downregulated in TCGA analysis. However, there was a significant upregulation of EEF1E1 mRNA levels in tumor tissues (Fig 4).

#### Prognostic significance

To ascertain the prognostic value of these factors in gastric cancer, OS and FP (only these two parameters available) were determined in high mRNA expression and low mRNA expression group (S8 Table). Higher mRNA levels of EEF1A2 and EEF1G predicted poor OS and FP, while elevated transcript levels of EEF1B2 and EEF1E1 predicted better OS and FP. Expression levels of other translation factors did not show any correlation with survivability of patients. Keeping in mind the importance of Her2 status of gastric cancer patients in devising treatment therapy, correlation of expression levels of genes with survival outcome was investigated in different tumor stages and Her2 status. As shown in S9 Table, higher EEF1A2 levels correlated with worse survival outcome in stage II and III only. Higher EEF1B2 levels correlated with better OS in all tumor stages. Interestingly, association of expression with survival outcome was lost for most translation factors in Her2+ group except, EEF1A2 and EEF2. EEF1A1 on the other hand, predicted better FP in Her2+ group only.

### Expression levels and prognostic significance of elongation factors in brain and CNS cancer

Brain tumors can be classified as primary or secondary. Gliomas, meningiomas, pituitary adenomas and nerve sheath tumors constitute the most common primary brain tumors in that order. They are further classified, depending upon the tissue of origin, into astrocytoma, glioblastoma, meningioma, medulloblastoma, eppendymoma and oligodendroglioma.

In stark contrast to the observations made in breast and lung cancers, Oncomine analysis showed that EEF1A1 transcript levels were significantly elevated in brain and CNS cancers. In two datasets of Shai’s studies, EEF1A1 was significantly upregulated, in oligodendroglioma and astrocytoma. In Bredel’s dataset, two analyses showed that EEF1A1 mRNA levels were significantly upregulated in anaplastic dendroglioma and oligodendroglioma. Interestingly, for EEF1A2, significantly reduced levels in tumor were observed in four unique analyses. As per Pomeroy’s datasets, EEF1A2 displayed very high downregulation in malignant glioma, classic medulloblastoma, and in desmoplastic medulloblastoma. Similarly, in Sun’s study, EEF1A2 was quite evidently under-expressed in glioblastoma, compared to normal tissue.

For EEF1B2, significantly elevated levels were observed in atypical teratoid/rhabdoid tumor and oligodendroglioma in Pomeroy’s dataset. EEF1G mRNA levels were significantly upregulated in five unique analyses from three different datasets (Pomeroy’s, Rickman’s and French’s). The tumor subtypes wherein upregulation was observed included desmoplastic medulloblastoma, oligodendroglioma, rhabdoid tumor, astrocytoma and classic medulloblastoma. EEF1D was overexpressed in Rickman’s study for astrocytoma and also in TCGA dataset of Glioblastoma. As for EEF1E1, two analyses in Shai’s datasets showed that it was upregulated in astrocytoma and oligodendroglioma. Similarly for EEF2, Pomeroy’s datasets exhibited increased transcript levels in desmoplastic medulloblastoma, classic medulloblastoma and rhabdoid tumor. All the above results are summarized in S10 Table.

**Fig 5:**
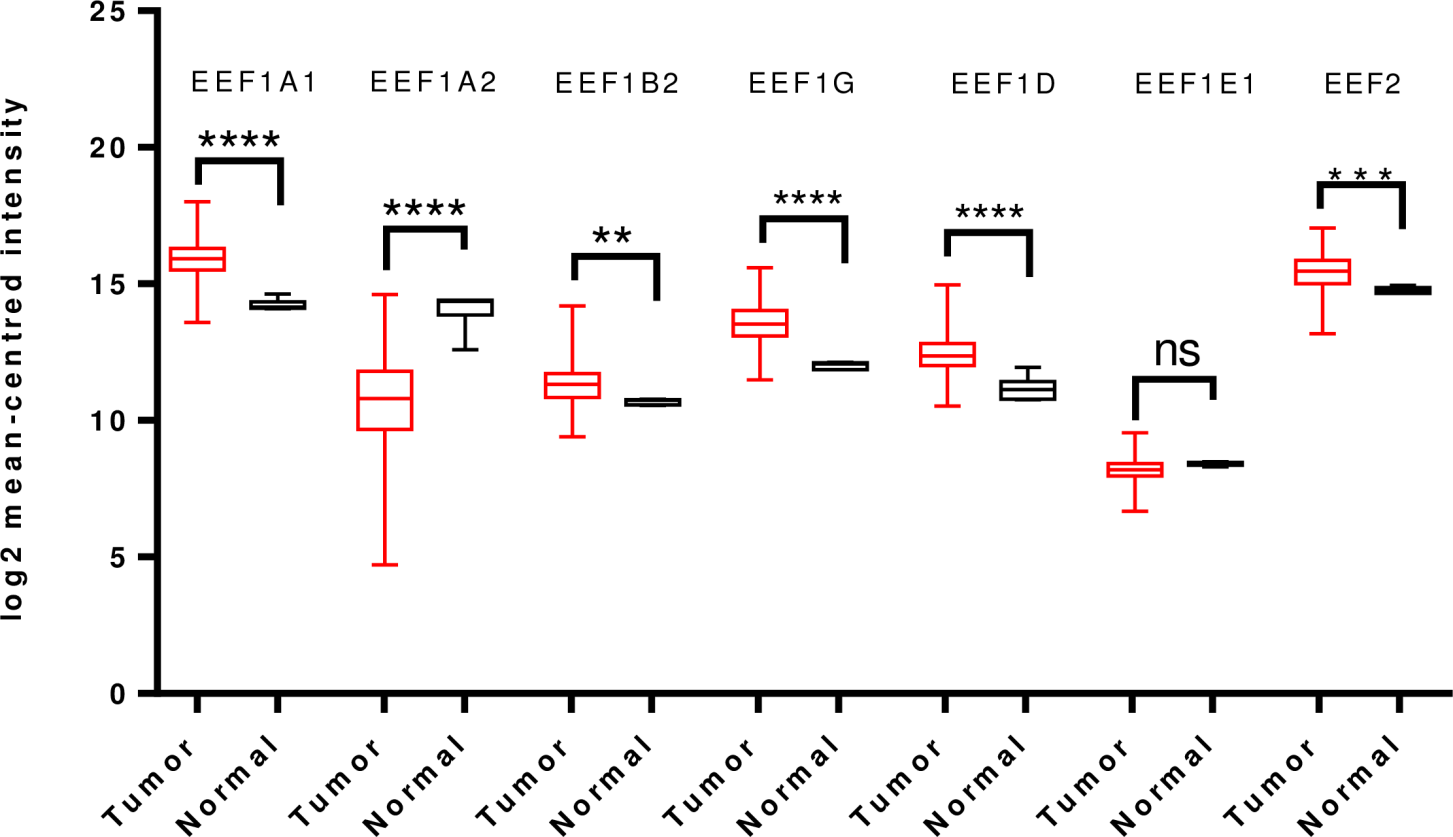
Analysis of mRNA expression levels of elongation factors in glioblastoma and gliomas using TCGA mRNA HiSeq expression data. Box-whisker plots indicate the differences in transcript levels of each elongation factor, between normal and tumor samples. The median value is represented by the middle line in the boxes. Statistical differences were ascertained by two-tailed Student’s t-test using graph-pad prism software. ****p ≤ 0.0001, ***p ≤ 0.001, ** p ≤ 0.01, ns–non significant.

In line with Oncomine analysis, TCGA analyses were carried out. Glioblastoma and glioma dataset revealed that EEF1A1, EEF1G, EEF1D and EEF2 were significantly overexpressed in tumor tissues whereas EEF1A2 was significantly downregulated. No significant difference in EEF1E1 expression was observed, in cancer tissue (Fig 5).

#### Prognostic significance

We carried out the correlation analysis between the expression levels of elongation factors and survival outcome of glioblastoma and glioma patients, using SurvExpress database. EEF1A1 expression was observed to be significantly upregulated in patients belonging to the low-risk group compared to the high risk group, and predicted better survivability. Interestingly, EEF1A2 transcript levels were also higher in low risk-group patients; however, no significant difference in survival was seen. Likewise, we found significantly higher mRNA levels of EEF1B2, EEF1G, EEF1D, EEF1E1 and EEF2 in low-risk group patients, which accordingly predicted favourable survival outcome in the group (S1 Fig).

### Expression levels and prognostic significance of elongation factors in prostate cancer

Prostate cancer has various histological types, but occurs mostly in the gland cells, accounting for 99% of the total reported cases. It comprises of acinar adenocarcinoma, ductal adenocarcinoma, transitional cell cancer, squamous cell cancer, small cell prostate cancer among others. In the Oncomine database, no significant difference was observed in mRNA levels of EEF1A1, between prostate carcinoma and normal tissue. However, for EEF1A2, Magee’s and LaTulippe’s datasets revealed overexpression of the gene. For EEF1B2, no study satisfied the statistical thresholds for significance. According to Singh’s dataset, EEF1G, EEF1D, EEF1E1 and EEF2 transcript levels were significantly upregulated. Furthermore, increased expression of EEF1E1 in prostate carcinoma was indicated in Tomlin’s dataset as well. All the results are summarized in S11 Table.

**Fig 6:**
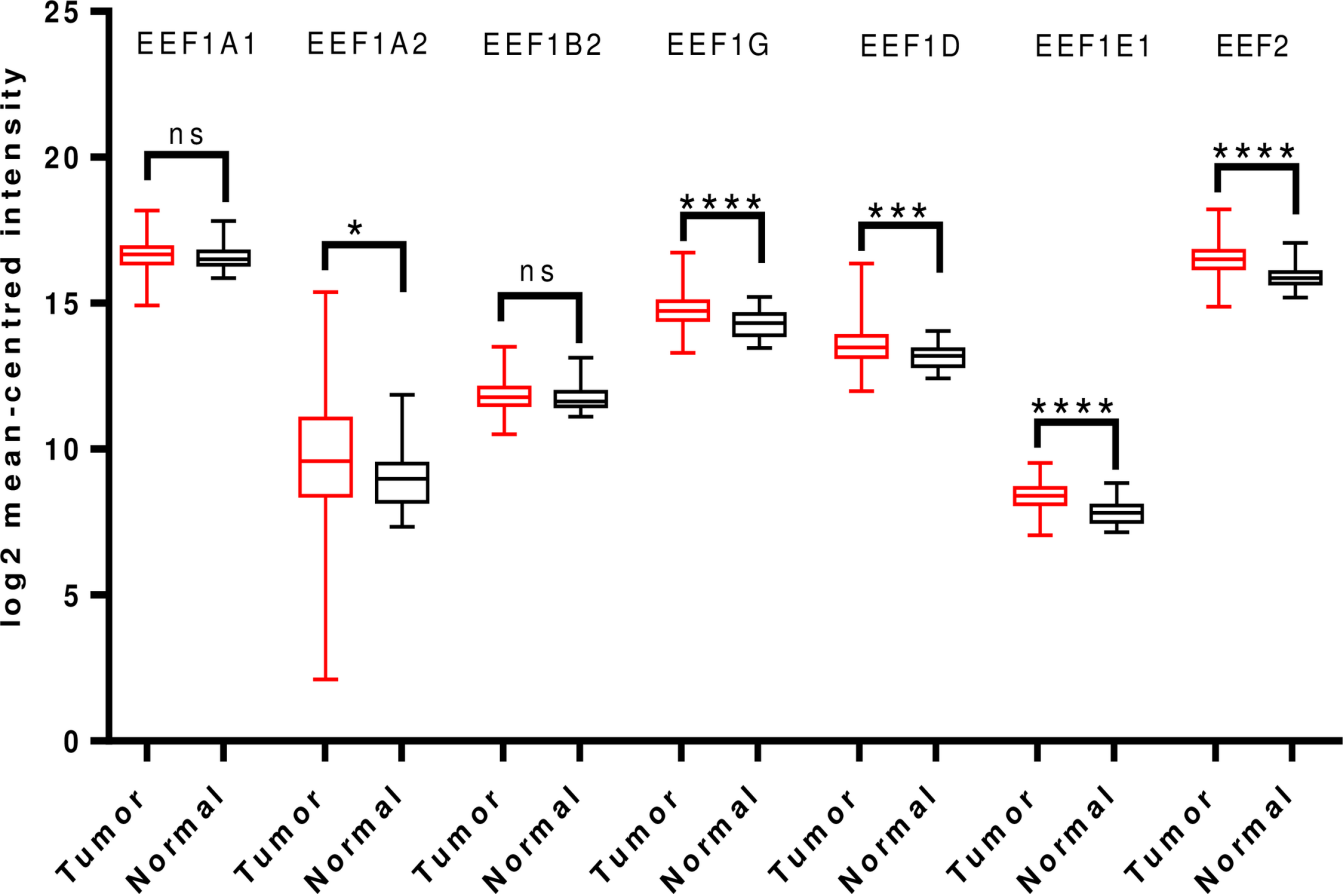
Analysis of mRNA expression levels of elongation factors in prostate cancer utilizing TCGA mRNA HiSeq expression data. Box-whisker plots display the differences in transcript levels of each elongation factor between normal and tumor samples. The median value is represented by the middle line in the boxes. Statistical differences were ascertained by two-tailed Student’s t-test using graph-pad prism software. ****p ≤ 0.0001, ***p ≤ 0.001, *p ≤ 0.05, ns–non significant.

Analysis of HiSeq expression data from TCGA dataset revealed no significant difference in transcript levels of EEF1A1 and EEF1B2. However, significant overexpression of EEF1A2, EEF1G, EEF1D, EEF1E1 and EEF2 was seen in tumor tissues (Fig 6).

#### Prognostic significance

SurvExpress analysis was used for ascertaining correlations between the expression of the genes with survival, in prostate cancer. High-risk group patients had higher EEF1A1, EEF1B2, EEF1G, EEF1E1 expression, although the difference between survival outcomes of the two groups was not significant. On the other hand, reduced EEF1A2 and EEF1D expression was present in the high-risk group, but again, no significant difference was seen, between survival outcomes of the two groups. Nevertheless, significantly worse survival outcome was associated with higher expression of EEF2 (S2 Fig).

### Expression levels and prognostic significance of elongation factors in colorectal cancer

Most of the colon and rectal cancers are of adenocarcinoma subtype (95% of all cases). Mucinous adenocarcinoma (10–15% of all cases) and signet ring cell adenocarcinoma (<1% of all cases) are less common subtypes of colon adenocarcinoma. Other types of colorectal cancers, which are encountered rarely, include primary colorectal lymphomas, gastrointestinal stromal tumors (GISTs), leiomyosarcomas and melanomas.

Oncomine analysis of colorectal cancer with the pre-specified thresholds didn’t return any datasets with significant difference in mRNA levels, for EEF1A1, EEF1A2, EEF1B2, EEF1G and EEF2. However, a single unique analysis of Hong’s dataset showed that EEF1D was significantly upregulated in colorectal carcinoma. EEF1E1 transcript levels on the other hand, were significantly upregulated in four different datasets (Skrzypczak, Kaiser, Hong, Skrzypczak) for rectal mucinous adenocarcinoma subtype (S12 Table).

**Fig 7:**
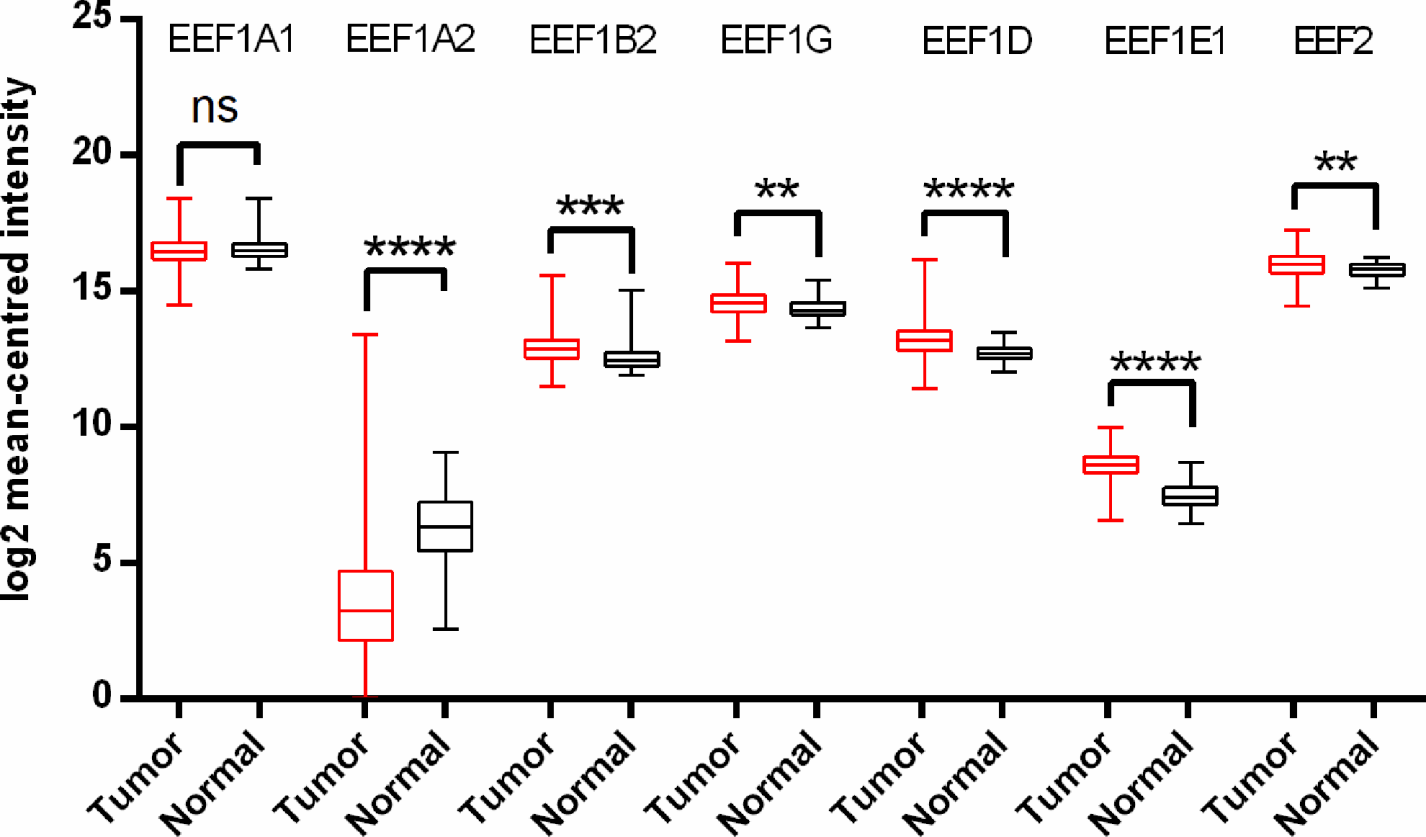
Analysis of mRNA expression levels of elongation factors in colorectal cancer utilizing TCGA mRNA HiSeq expression data. Box-whisker plots illustrate the differences in transcript levels of each elongation factor, between normal and tumor samples. The median value is represented by the middle line in the boxes. Statistical differences were ascertained by two-tailed Student’s t-test using graph-pad prism software. ****p ≤ 0.0001, ***p ≤ 0.001, ** p ≤ 0.01, *p ≤ 0.05, ns–non significant.

TCGA dataset revealed no significant difference of EEF1A1 mRNA levels, between tumor and normal group. EEF1A2 was significantly downregulated in the colorectal cancer group, compared to normal. Rest of the translation factors under study, viz. EEF1B2, EEF1G, EEF1D, EEF1E1 and EEF2, presented increased transcript levels in tumor group compared to normal, the most prominent being EEF1E1 (Fig 7).

#### Prognostic significance

SurvExpress analysis revealed significantly reduced mRNA levels of EEF1A1, EEF1B2, EEF1E1, EEF1G and EEF2 in the high-risk group, in colorectal cancer and, predicted poor survival. On the contrary, higher levels of EEF1A2 were observed in high-risk group and predicted low survival. EEF1D expression was also elevated in high-risk group patients, but showed no significant difference in survivability between the two groups (S3 Fig).

### Expression levels and prognostic significance of elongation factors in lymphoma

Lymphomas originate in B cells, T cells, or NK cells in the lymphatic system. Non-Hodgkin’s lymphoma (NHL) comprises three major subtypes viz. B-cell lymphoma (90% of all cases), T-cell lymphoma (about 10% of all cases) and NK cell lymphoma (<1% of all cases). Other common subtypes of B-cell lymphoma include diffuse large B-cell lymphoma, follicular lymphoma, mantle cell lymphoma, small lymphocytic lymphoma, primary mediastinal large B-cell lymphoma, splenic marginal zone B-cell lymphoma, primary effusion lymphoma, and Burkitt lymphoma/Burkitt cell leukaemia. Subtypes of T-cell and NK-cell lymphoma include anaplastic large cell lymphoma, systemic type peripheral T-cell lymphoma and, angioimmunoblastic T-cell lymphoma.

Oncomine analysis revealed that a number of elongation factors show differential expression in lymphomas as well (S13 Table). EEF1A1 was significantly downregulated in angioimmunoblastic T-Cell Lymphoma, centroblastic lymphoma and marginal zone B-Cell lymphoma subtypes, in Piccaloga’s, Bassos’s and Storz’s datasets, respectively. However, Basso’s dataset for follicular lymphoma revealed upregulation of EEF1A1. EEF1A2 expression did not show any differential expression in tumors. For EEF1B2, three studies from Brune’s dataset showed elevated transcript levels in follicular lymphoma, diffuse large B-Cell lymphoma and, in Burkitt's lymphoma. Likewise for EEF1G, significant overexpression of the gene was observed in Brune’s dataset for Burkitt's lymphoma and diffuse large B-Cell Lymphoma. Quite notably, EEF1D was significantly overexpressed in ten unique analyses, across different lymphoma subtypes (anaplastic large cell lymphoma, ALK-negative, anaplastic large cell lymphoma, ALK-Positive, classical Hodgkin's lymphoma, acute adult T-cell leukaemia/lymphoma, Burkitt's lymphoma, Hodgkin's lymphoma, follicular lymphoma, activated B-cell-like diffuse large B-cell lymphoma, diffuse large B-cell lymphoma, germinal center B-Cell-like diffuse large B-cell lymphoma), in four different datasets (Eckerle, Choi, Brune, Compagno). EEF1E1 mRNA was overexpressed in two datasets, i.e. Basso’s for Burkitt's lymphoma and, Brune’s for diffuse large B-Cell lymphoma. Contrariwise, it was downregulated in marginal zone B-Cell lymphoma in Storz dataset. Similarly, reduced transcript levels of EEF2 were observed in follicular lymphoma and T-Cell/histiocyte-rich large B-Cell lymphoma, in Eckerle’s dataset, as opposed to overexpression in anaplastic large cell lymphoma in the same dataset. TCGA analysis and survival analysis in lymphoma could not be done due to unavailability of data.

### Expression levels and prognostic significance of elongation factors in ovarian cancer

More than 30 different types of ovarian cancer have been typified, primarily depending upon the type of cell from which they start. Cancerous ovarian tumors start from three common cell types. Epithelial tumors comprise 90% of all cases, while stromal carcinoma tumors and germ cell carcinoma tumors constitute 5% of all cases. Small cell carcinoma of the ovary is the rarest and highly malignant type of ovarian cancer. Majority of epithelial ovarian tumors, including serous adenomas, mucinous adenomas and Brenners tumors are benign in nature while malignant ones are carcinomas. Oncomine analysis for ovarian cancer revealed significant difference of mRNA levels between tumor and normal samples, for EEF1A1, EEF1A2 and EEF1E1 only. While EEF1A1 was found to be downregulated in ovarian serous surface papillary carcinoma in Welsh’s dataset, EEF1A2 was upregulated in ovarian clear cell adenocarcinoma in Hendrix’s dataset. EEF1E1 mRNA levels were upregulated in ovarian serous adenocarcinoma in Yoshivara’s study (S14 Table). TCGA database didn’t contain data for normal ovarian samples, hence comparison of mRNA levels between tumor and normal samples could not be done. To ascertain the prognostic value of these factors in ovarian cancer, OS, PFS and PPS were determined for each gene (S15 Table). Higher expression of EEF1G predicted better OS and PFS in ovarian cancer patients. Apart from this, expression levels of other factors didn’t show any significant correlation with survival parameters.

### Expression levels and prognostic significance of elongation factors in pancreatic cancer

**Fig 8:**
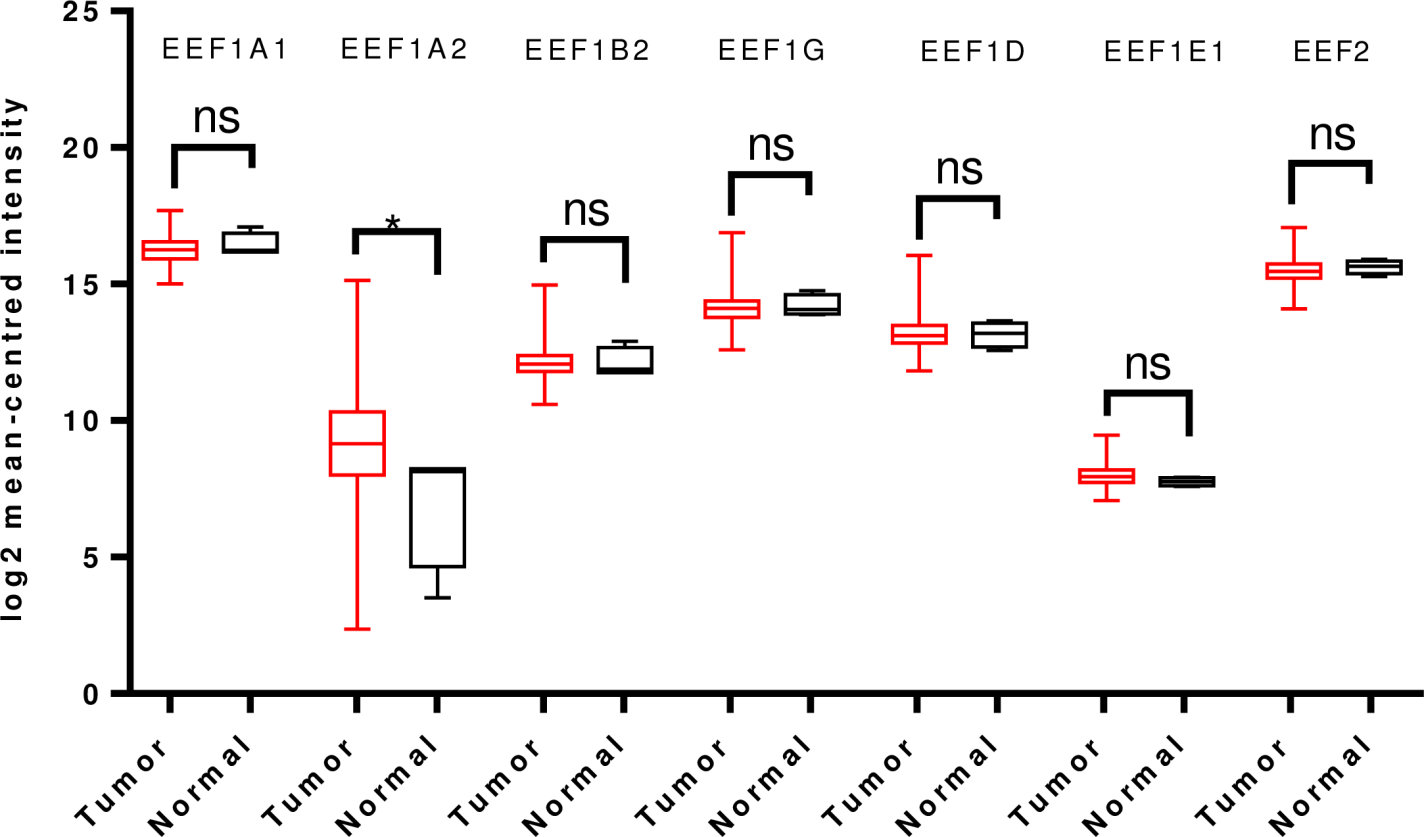
Analysis of mRNA expression levels of elongation factors in pancreatic cancer using TCGA mRNA HiSeq expression data. Box-whisker plots show the differences in transcript levels of each elongation factor between normal and tumor samples. The median value is represented by the middle line in the boxes. Statistical differences were ascertained by two-tailed Student’s t-test using graph-pad prism software. *p ≤ 0.05, ns–non significant.

Pancreatic cancer consists of two major types, i.e. tumors of exocrine gland and tumors of endocrine gland. Tumors of the exocrine glands are called adenocarcinomas which constitute 95% of total cancer types and, form in the pancreatic ducts. The other categories of endocrine type are less common and most often benign. These tumors are otherwise called islet cell tumors or neuroendocrine tumors. In pancreatic cancer, the mRNA levels of EEF1A1, EEF1A2, EEF1B2, EEF1D and EEF1E1 were found to be significantly downregulated, as shown by Oncomine analysis (S14 Table). Since the datasets analysed in Oncomine had very small sample size, hence TCGA analysis was carried out to further investigate the same. It revealed that while mRNA levels of other translation factors were not significantly different between tumor and normal samples, EEF1A2 mRNA levels were comparatively higher in tumor samples (Fig 8). Moreover, SurvExpress analysis found that EEF1A1 mRNA levels were higher in the low-risk group but not significantly associated with survivability. High expression levels of EEF1A2 and EEF1G predicted poor survivability of patients in the high-risk group, whereas lower expression of EEF1B2, EEF1E1 and EEF2 predicted better survival. Higher EEF1D levels in the high-risk group did not correlate with any difference in survivability between the risk groups (S4 Fig).

### Expression levels and prognostic significance of elongation factors in kidney cancer

**Fig 9:**
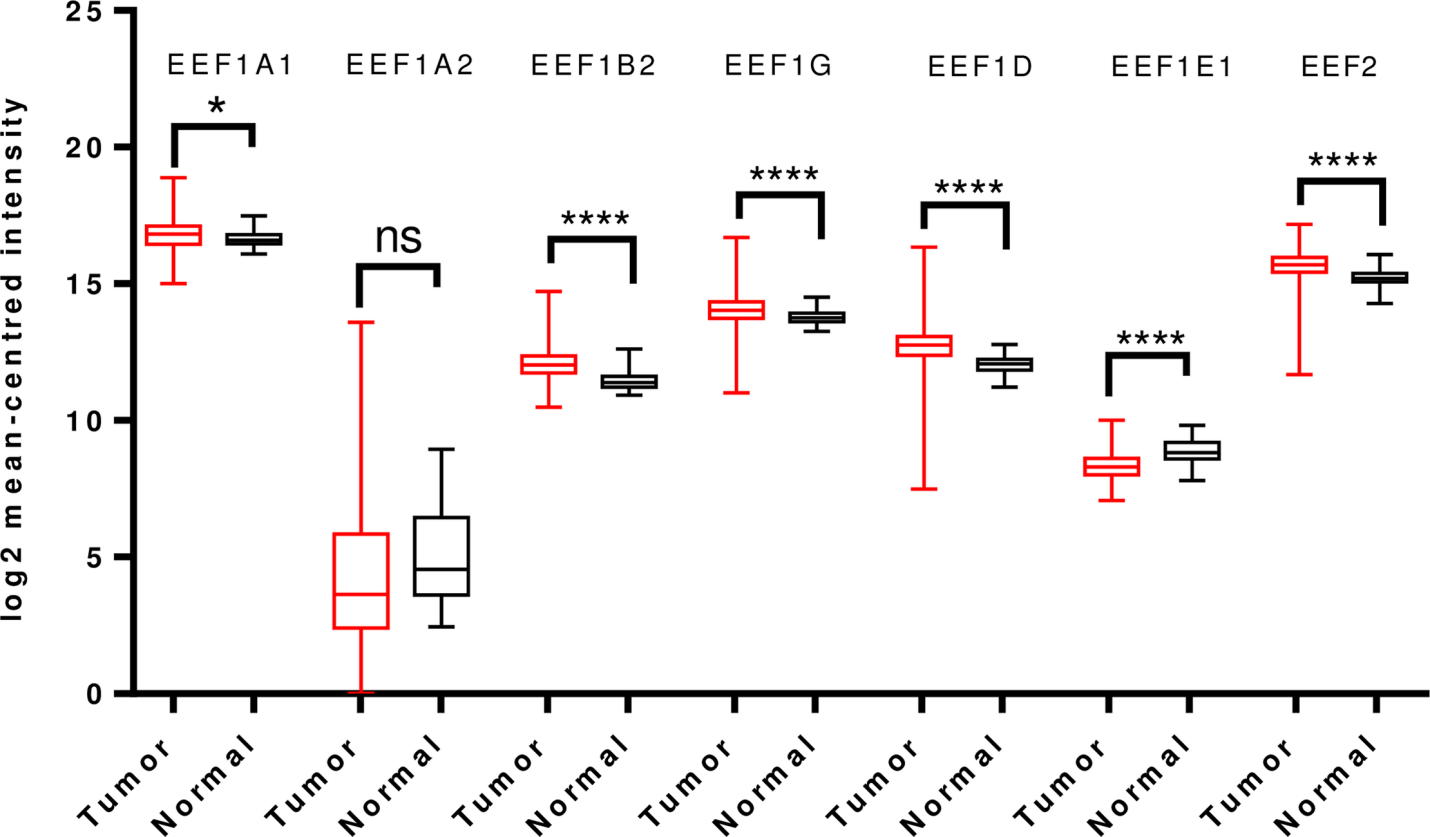
Analysis of mRNA expression levels of elongation factors in kidney cancer (clear cell carcinoma) cancer using TCGA mRNA HiSeq expression data. Box-whisker plots display the differences in transcript levels of each elongation factor, between normal and tumor samples. The median value is represented by the middle line in boxes. Statistical differences were ascertained by two-tailed Student’s t-test using graph-pad prism software. ****p ≤ 0.0001, *p ≤ 0.05, ns–non significant.

Renal cell carcinoma is the most common type of kidney cancer, accounting for 85% of the total diagnosis. The next most frequent type is transitional cell carcinoma and other types include sarcoma, Wilms tumor and lymphoma. The most common type of kidney cancer cells (classification as per cell type) are clear cell, papillary, sarcomatoid, medullary, chromophobe, oncocytoma and angiomyolipoma. Oncomine analysis of kidney cancer revealed that, EEF1A2 and EEF1B2 mRNA levels were upregulated in cancer samples. For EEF1D, two analyses of Cutcliffe’s dataset showed higher mRNA levels in renal Welms tumor and clear cell carcinoma. However, a single analysis from Gumz dataset showed a lower EEF1D transcript level in clear cell carcinoma. EEF1E1 was found to be downregulated in chromophobe renal cell carcinoma compared to normal tissue, in Yuseknko’s dataset (S14 Table). As per TCGA analysis, expression levels of EEF1A1, EEF1B2, EEF1G, EEF1D and EEF2 were significantly upregulated in kidney clear cell carcinoma while that of EEF1E1 was downregulated (Fig 9).

SurvExpress analysis using TCGA-KIRC (Kidney clear cell carcinoma) dataset showed that higher expression levels of EEF1A1, EEF1G and EEF2 predicted better survival in low-risk group patients. Higher expression of EEF1A2, EEF1D and EEF1E1 in high-risk group patients did not predict significant difference in survivability between the two groups (S5 Fig).

### Expression levels and prognostic significance of elongation factors in head and neck cancers

**Fig 10:**
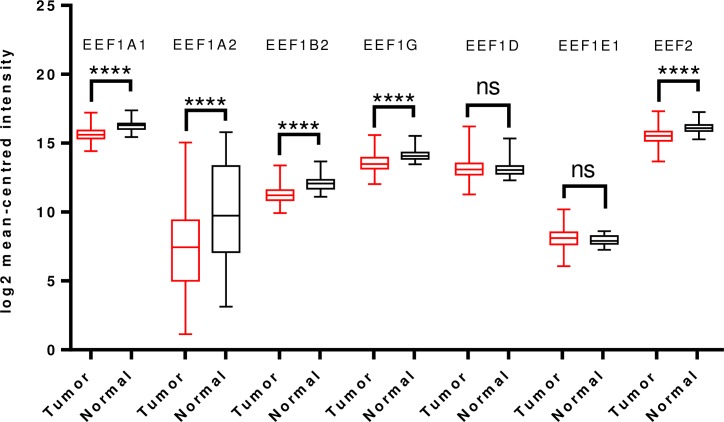
Analysis of mRNA expression levels of elongation factors in head and neck cancer using TCGA mRNA HiSeq expression data. Box-whisker plots depict the differences in transcript levels of each elongation factor between normal and tumor samples. The median value is represented by the middle line in the boxes. Statistical differences were ascertained by two-tailed Student’s t-test using graph-pad prism software. ****p ≤ 0.0001, ns–non significant.

Cancers that usually begin in the squamous cells that line the moist, mucosal surfaces inside the head and neck are collectively called as head and neck cancers. They are further categorised into different types depending upon the part of the head or neck from where they originate. These areas are demarcated as oral cavity, pharynx, larynx, paranasal sinuses, nasal cavity and, salivary glands. For tumors of head and neck type, Oncomine analysis revealed that mRNA levels of EEF1A2 and EEF1B2 were significantly lower in tongue squamous cell carcinoma, salivary gland adenoid cystic carcinoma and hypopharyngeal squamous cell carcinoma, respectively. EEF1D levels were found to be upregulated in cancer tissue, in two analyses of Pyeon’s multi-cancer dataset (S14 Table). TCGA analysis further confirmed that mRNA levels of EEF1A1, EEF1A2, EEF1B2, EEF1G and EEF2 were significantly downregulated in tumor tissues than normal (Fig 10).

SurvExpress analysis showed that expression levels of EEF1A1, EEF1A2, EEF1B2, EEF1G and EEF1E1 were significantly higher in the high-risk group, whereas those of EEF1D and EEF2 were higher in the low-risk groups. However, no statistically significant difference was observed between the survivability of both the risk groups for any of the genes (S6 Fig).

### Expression levels and prognostic significance of elongation factors in liver cancer

**Fig 11:**
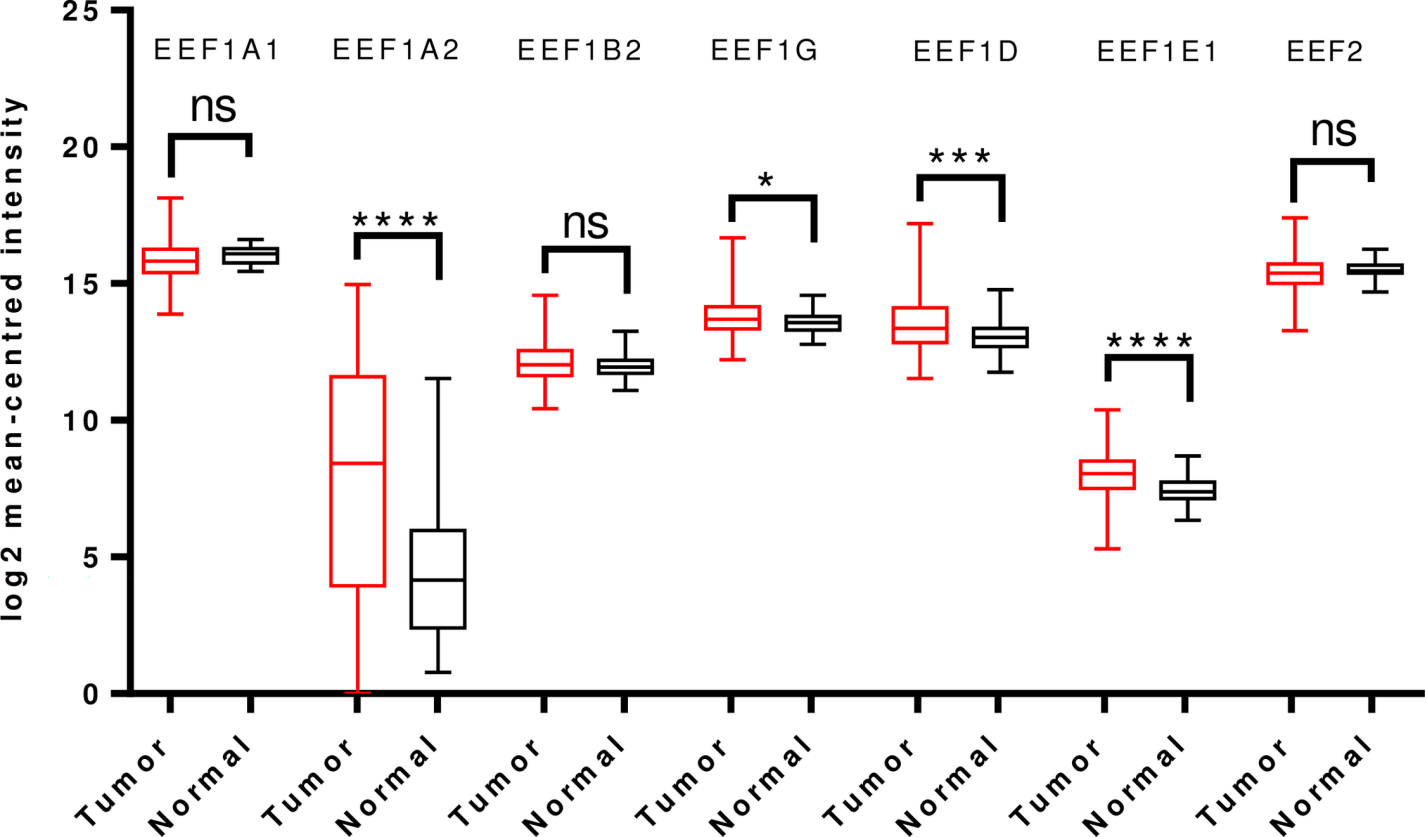
Analysis of mRNA expression levels of elongation factors in liver cancer using TCGA mRNA HiSeq expression data. Box-whisker plots showing the differences in transcript levels of each elongation factor between normal and tumor samples. The median value is represented by the middle line in the boxes. Statistical differences were ascertained by two-tailed Student’s t-test using graph-pad prism software. ****p ≤ 0.0001, ***p ≤ 0.001, *p ≤ 0.05, ns–non significant.

Liver cancer is classified into different types depending upon the type of cells involved. Hepatocellular carcinoma (HCC) is the most common type (75% of all liver cancers). Other types include fibromellar HCC, cholangiocarcinoma (bile duct cancer) and angiosarcoma. For liver cancer, Oncomine analysis revealed that transcript levels of only EEF1E1 were significantly elevated in tumor tissues in Roessler’s datasets. Other factors did not show any significant difference between tumor and normal groups (S14 Table). However TCGA analysis revealed that in addition to EEF1E1, mRNA levels of EEF1A2, EEF1G, and EEF1D are also significantly upregulated in tumor tissues (Fig 11).

SurvExpress analysis was done to determine prognostic value of the genes in liver cancer. Higher expression levels of EEF1A1, EEF1B2, EEF1G, EEF1E1 and EEF2 predicted worse survival in high-risk group (S7 Fig).

### Discussion

Due to scarcity of studies investigating perturbations of translation elongation factors in cancers and subsequent roles in the initiation and progression of tumorigenesis, we carried out a comprehensive data mining analysis of the expression levels of seven translation elongation factors. Some of these factors have been reported to have differential expression in one or more cancers, by a limited number of studies [19–28]. For other elongation factors, the published literature does not provide sufficient information to assign a favourable or unfavourable role in the initiation or progression of different cancers.

Overall, our analysis has summarised the expression profile of different elongation factors across cancer types and determined their prognostic importance. Our consistent finding is a higher expression of EEF1A2 (breast, lung, prostate, pancreatic, liver) and reduced expression of EEF1A1 (breast, lung, gastric, kidney, head and neck) in most of the cancers, which is in accordance with previously reported findings from perturbation of EEF1A2, showing it as an oncogene in different cancers [7–9, 34, 40]. Previous studies have highlighted the mutually exclusive expression of either one of the two variants (EEF1A1 and EEF1A2); wherein the eEF1A2 variant is expressed only in the mature, terminally differentiated neurons and muscles [41]. Furthermore, the loss of EEF1A1 isoform during development in specialised tissues occurs in parallel with the rise in EEF1A2 levels in the same [42, 43]. We found that there is a reciprocal relationship between EEF1A1 and EEF1A2 transcript levels in tumor tissues of different cancer types, indicating towards a tight regulation of any one isoform over another, in cancers also. In addition to EEF1A2, our findings support the notion that EEF1A1 might also play a pro-tumorigenic role in progression of certain cancers such as liver cancer, kidney cancer, gliomas and glioblastomas, although its expression seems to be a predictor of good prognosis in most of the cancers (breast, lung, brain, kidney). EEF1A1 may be predictor of good prognosis, not due to the direct involvement of EEF1A1 imparting anti-tumor activities, rather the correlation of its higher expression levels with lower levels of the proto-oncogenic isoform EEF1A2, thereby serving as a biomarker indirectly. It is noteworthy that both of these isoforms have been known to play opposing roles with regards to apoptosis. While EEF1A1 has been widely reported as a pro-apoptotic protein [11–14, 20], EEF1A2 is mostly cited to have anti-apoptotic role [5, 8].

We also found that the previously reported pro-tumorigenic roles of EEF1G and EEF1D [20–24] can be extended to other cancers viz. colorectal, liver, kidney, prostate and brain cancer, wherein elevated levels of both proteins are seen. However, neither the expression of these proteins nor their prognostic significance is consistent, in all the cancers. A previous report has cited EEF1E1 as a tumor suppressor gene in gastric and colon adenocarcinoma [25]. We also found that higher EEF1E1 expression was related to better survival in these two cancer types, however in many other cancer types (breast, lung, gastric, prostate, colorectal, liver) EEF1E1 was found to be overexpressed in tumor tissues and predicted poor survival (breast, lung, liver). Reason behind the stark contrast observations in cancer specific manner, needs further investigation. Earlier studies have hinted at the pro-tumorigenic role of EEF1B2 and EEF2 in cancers [19, 26–28] which was evident in the present study also.

We further extended our analysis to different histological subtypes and tumor stages in breast, lung and gastric cancer. Interestingly, we saw that with the sub-intrinsic classification of breast tumors, the prognostic significance of the factors increases preferentially in particular subtypes. For example, the higher EEF1A1 expression which is otherwise a biomarker for good prognosis overall, becomes an indicator for poor overall survival in basal subtype. In lung cancer, we saw that expression of elongation factors was more strongly associated with survival in adenocarcinoma subtype and stage I tumors. Likewise, we see that survival outcomes in gastric cancer were more strongly associated with expression levels of elongation factors in Her2 negative group. This suggests the involvement of these proteins in specific intrinsic subtypes of different cancers.

Overexpression of most factors predicted a poor prognosis in breast (EEF1D, EEF1E1, and EEF2) and lung cancer (EEF1A2, EEF1B2, EEF1G, EEF1E1) in KM analysis. SurvExpress analysis revealed that the transcript levels of these factors were mostly higher in the high-risk group patients than in the low-risk group, only in specific type of cancers. Overall, we see that in addition to EEF1A2; EEF1G, EEF1D, EEF1E1 and EEF2 are significantly upregulated in different cancer types and are associated with better and worse survival outcome in specific cancers. Future studies are required to ascertain the precise roles of these elongation factors in tumor progression. It is quite possible that like EEF1A2, other translation factors discussed herein could also be promising therapeutic targets and prognostic biomarkers in human cancers as most of them are seen to overexpressed in a wide variety of cancers.

Previous studies have noted that multiple elongation factors are encoded by terminal oligopyrimidine (TOP) mRNA’s and show a growth associated translation regulation [44]. Stimulation of protein synthesis in response to growth factors is well studied [45–47]. Even though previous studies have documented the aberrant mRNA expression pattern of translation factors in different cancers, the factors responsible for it remain largely unknown. Increased transcript levels of the genes can be attributed to gene amplification and/or transcription rates. EEF1A1, EEF1A2 and EEF1D have previously been reported to be potential candidates for gene amplification, leading to observed overexpression in certain cancers [24, 48, 49]. Apart from gene amplification, the transcriptional regulation of these eukaryotic translation factors in different cancers is not well-studied. A single study has reported the regulation of EEF1α mRNA levels by EGF family of growth factors [50] EEF1A2 mRNA levels are elevated in ERα+ breast cancers [51, 52], indirectly indicating the involvement of estrogen signalling pathway in its transcriptional regulation. In absence of much information on the regulation of expression of transcription factors, we decided to use bioinformatics analysis. We observed that the promoter sequences of these translation elongation factor genes harbour binding sites for transcription factors which are commonly deregulated in the pathogenesis of human cancers, such as cFos, cJun, c-Ets1, Sp1, Sp3, Pax2 and, Pax6 among others (S8 Fig). It is noteworthy that SP1 site has already been reported to be essential for heregulin-β1 stimulation of EF-1α promoter [50].

In conclusion, the key observations we made were the reciprocal expression of EEF1A isoforms in different cancers which might indicate that a ratio of both isoforms in different tissues could be a possible driver of tumorigenesis. In addition to EEF1A2, other translation factors EEF1B2, EEF1G, EEF1D, EEF1E1, and EEF2 were also found to be frequently overexpressed in different cancers. We found non-uniform pattern of correlation of these factors with survival parameters across different cancer. In this regard, further *in-vitro* and *in-vivo* perturbation studies could shed light on the role of non-canonical roles of these factors in initiating carcinogenesis. How the transcriptional rate of these elongation factors is dysregulated during cancer progression, is still an open question.

## Supporting information

S1 Fig SurvExpress analysis of effect of elongation factors expression on survival in glioblastoma and glioma TCGA dataset (GBMLGG). Upper panel shows the Kaplan-Meier curve of risk groups for each gene. The concordance index and p-value of log-rank testing equality of survival curves are indicated. The X-axis represents the time (days) of the study. The number of samples not presenting the event at the matching time has been shown in rows with corresponding color. Lower panel shows the box plots indicating the difference in expression of gene between risk groups, p-values are derived from t-test between both groups. The Y-axis shows the expression levels. In both panels, green color shows low risk group and red color shows high risk group.
(TIF)

S2 Fig SurvExpress analysis of effect of elongation factors expression on survival in prostate cancer TCGA dataset (PRAD). Upper panel shows the Kaplan-Meier curve of risk groups for each gene. The concordance index and p-value of log-rank testing equality of survival curves are indicated. The X-axis represents the time (days) of the study. The number of samples not presenting the event at the matching time has been shown in rows with corresponding color. Lower panel shows the box plots indicating the difference in expression of gene between risk groups, p-values are derived from t-test between both groups. The Y-axis shows the expression levels. In both panels, green color shows low risk group and red color shows high risk group.
(TIF)

S3 Fig SurvExpress analysis of effect of elongation factors expression on survival in colorectal cancer TCGA dataset (COADREAD). Upper panel shows the Kaplan-Meier curve of risk groups for each gene. The concordance index and p-value of log-rank testing equality of survival curves are indicated. The X-axis represents the time (days) of the study. The number of samples not presenting the event at the matching time has been shown in rows with corresponding color. Lower panel shows the box plots indicating the difference in expression of gene between risk groups, p-values are derived from t-test between both groups. The Y-axis shows the expression levels. In both panels, green color shows low risk group and red color shows high risk group.
(TIF)

S4 Fig SurvExpress analysis of effect of elongation factors expression on survival in pancreatic cancer TCGA dataset (PAAD). Upper panel shows the Kaplan-Meier curve of risk groups for each gene. The concordance index and p-value of log-rank testing equality of survival curves are indicated. The X-axis represents the time (days) of the study. The number of samples not presenting the event at the matching time has been shown in rows with corresponding color. Lower panel shows the box plots indicating the difference in expression of gene between risk groups, p-values are derived from t-test between both groups. The Y-axis shows the expression levels. In both panels, green color shows low risk group and red color shows high risk group.
(TIF)

S5 Fig SurvExpress analysis of effect of elongation factors expression on survival in kidney cancer clear cell carcinoma dataset TCGA dataset. Upper panel shows the Kaplan-Meier curve of risk groups for each gene. The concordance index and p-value of log-rank testing equality of survival curves are indicated. The X-axis represents the time (days) of the study. The number of samples not presenting the event at the matching time has been shown in rows with corresponding color. Lower panel shows the box plots indicating the difference in expression of gene between risk groups, p-values are derived from t-test between both groups. The Y-axis shows the expression levels. In both panels, green color shows low risk group and red color shows high risk group.
(TIF)

S6 Fig SurvExpress analysis of effect of elongation factors expression on survival in head and neck cancer TCGA dataset (HNSC). Upper panel shows the Kaplan-Meier curve of risk groups for each gene. The concordance index and p-value of log-rank testing equality of survival curves are indicated. The X-axis represents the time (days) of the study. The number of samples not presenting the event at the matching time has been shown in rows with corresponding color. Lower panel shows the box plots indicating the difference in expression of gene between risk groups, p-values are derived from t-test between both groups. The Y-axis shows the expression levels. In both panels, green color shows low risk group and red color shows high risk group.
(TIF)

S7 Fig SurvExpress analysis of effect of elongation factors expression on survival in liver cancer TCGA dataset (LIHC). Upper panel shows the Kaplan-Meier curve of risk groups for each gene. The concordance index and p-value of log-rank testing equality of survival curves are indicated. The X-axis represents the time (days) of the study. The number of samples not presenting the event at the matching time has been shown in rows with corresponding color. Lower panel shows the box plots indicating the difference in expression of gene between risk groups, p-values are derived from t-test between both groups. The Y-axis shows the expression levels. In both panels, green color shows low risk group and red color shows high risk group.
(TIF)

S8 Fig Common transcription factors binding sites. Graphical representation of the common transcription factors binding sites, commonly present in the promoter sequences of all seven, eukaryotic translation elongation factor genes, with a dissimilarity margin less or equal to 15%. Binding site prediction that appear in all seven sequences are shown as boxes of different color and number, square bracket shows TRANSFAC accession ID.
(TIF)

S1 Table Differential expression analyses of elongation factors in breast cancer. (DOCX)

S2 Table Kaplan-Meier plotter data showing the correlation between different elongation factors and survival outcomes in breast cancer. Abbreviations: OS—Overall patient survival, FP—First progression, PPS—Post progression survival, DMFS—Distance metastasis free survival, RFS—Relapse free survival. p-values ≤ 0.05 were considered statistically significant and have been denoted in bold.
(DOCX)

S3 Table The correlation between elongation factors and survival outcomes in intrinsic subtypes of breast cancer. Abbreviations: OS: overall survival; RFS: relapse free survival; DMFS: distant metastasis free survival; PPS: post progression survival; HR: Hazard radio; CI: Confidence interval.
(DOCX)

S4 Table Differential expression analyses of elongation factors in lung cancer. (DOCX)

S5 Table Kaplan-Meier plotter data showing the correlation between different elongation factors and survival outcomes in lung cancer. Abbreviations: OS: overall survival; RFS: relapse free survival; DMFS: distant metastasis free survival; PPS: post progression survival; HR: Hazard radio; CI: Confidence interval.
(DOCX)

S6 Table The correlation between elongation factors and survival outcomes in lung cancer patients stratified by histological types and tumor stages. Abbreviations: OS: overall survival; RFS: relapse free survival; DMFS: distant metastasis free survival; PPS: post progression survival; HR: Hazard radio; CI: Confidence interval. p-values ≤ 0.05 were considered statistically significant and have been denoted in bold.
(DOCX)

S7 Table Differential expression analyses of elongation factors in gastric cancer. (DOCX)

S8 Table Kaplan-Meier plotter data showing the correlation between different elongation factors and survival outcomes in gastric cancer. Abbreviations: OS: overall survival; RFS: relapse free survival; DMFS: distant metastasis free survival; PPS: post progression survival; HR: Hazard radio; CI: Confidence interval. p-values ≤ 0.05 were considered statistically significant and have been denoted in bold.
(DOCX)

S9 Table The correlation between elongation factors and survival outcomes in gastric cancer patients stratified by tumor stages and Her2 status. Abbreviations: OS: overall survival; RFS: relapse free survival; DMFS: distant metastasis free survival; PPS: post progression survival; HR: Hazard radio; CI: Confidence interval. p-values ≤ 0.05 were considered statistically significant and have been denoted in bold.
(DOCX)

S10 Table Differential expression analyses of elongation factors in brain cancer. (DOCX)

S11 Table Differential expression analyses of elongation factors in prostate cancer. (DOCX)

S12 Table Differential expression analyses of elongation factors in colorectal cancer. (DOCX)

S13 Table Differential expression analyses of elongation factors in lymphoma. (DOCX)

S14 Table Differential expression analyses of elongation factors in other cancers. (DOCX)

S15 Table Kaplan-Meier plotter data showing the correlation between different elongation factors and survival outcomes in ovarian cancer. Abbreviations: OS: overall survival; RFS: relapse free survival; DMFS: distant metastasis free survival; PPS: post progression survival; HR: Hazard radio; CI: Confidence interval. p-values ≤ 0.05 were considered statistically significant and have been denoted in bold.
(DOCX)
