# Supplementary figures and images for: The expression profile and prognostic significance of eukaryotic translation elongation factors in different cancers
Source: PLoS One. 2018 Jan 17;13(1):e0191377. doi: 10.1371/journal.pone.0191377 (PMC5771626; doi:10.1371/journal.pone.0191377)

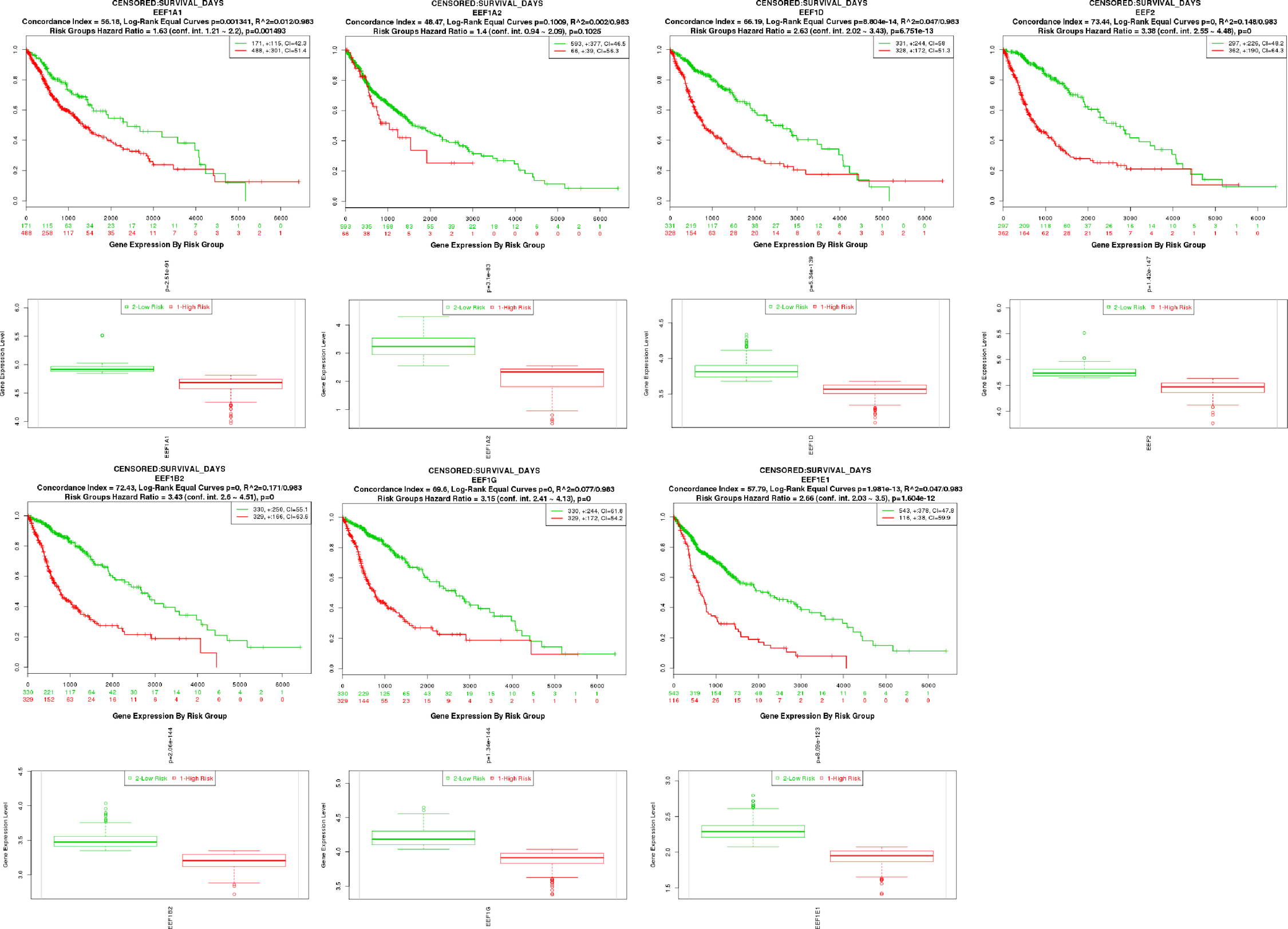

Supplement: S1 Fig — Upper panel shows the Kaplan-Meier curve of risk groups for each gene. The concordance index and p-value of log-rank testing equality of survival curves are indicated. The X-axis represents the time (days) of the study. The number of samples not presenting the event at the matching time has been shown in rows with corresponding color. Lower panel shows the box plots indicating the difference in expression of gene between risk groups, p-values are derived from t-test between both groups. The Y-axis shows the expression levels. In both panels, green color shows low risk group and red color shows high risk group. (TIF) [file pone.0191377.s001.tif]

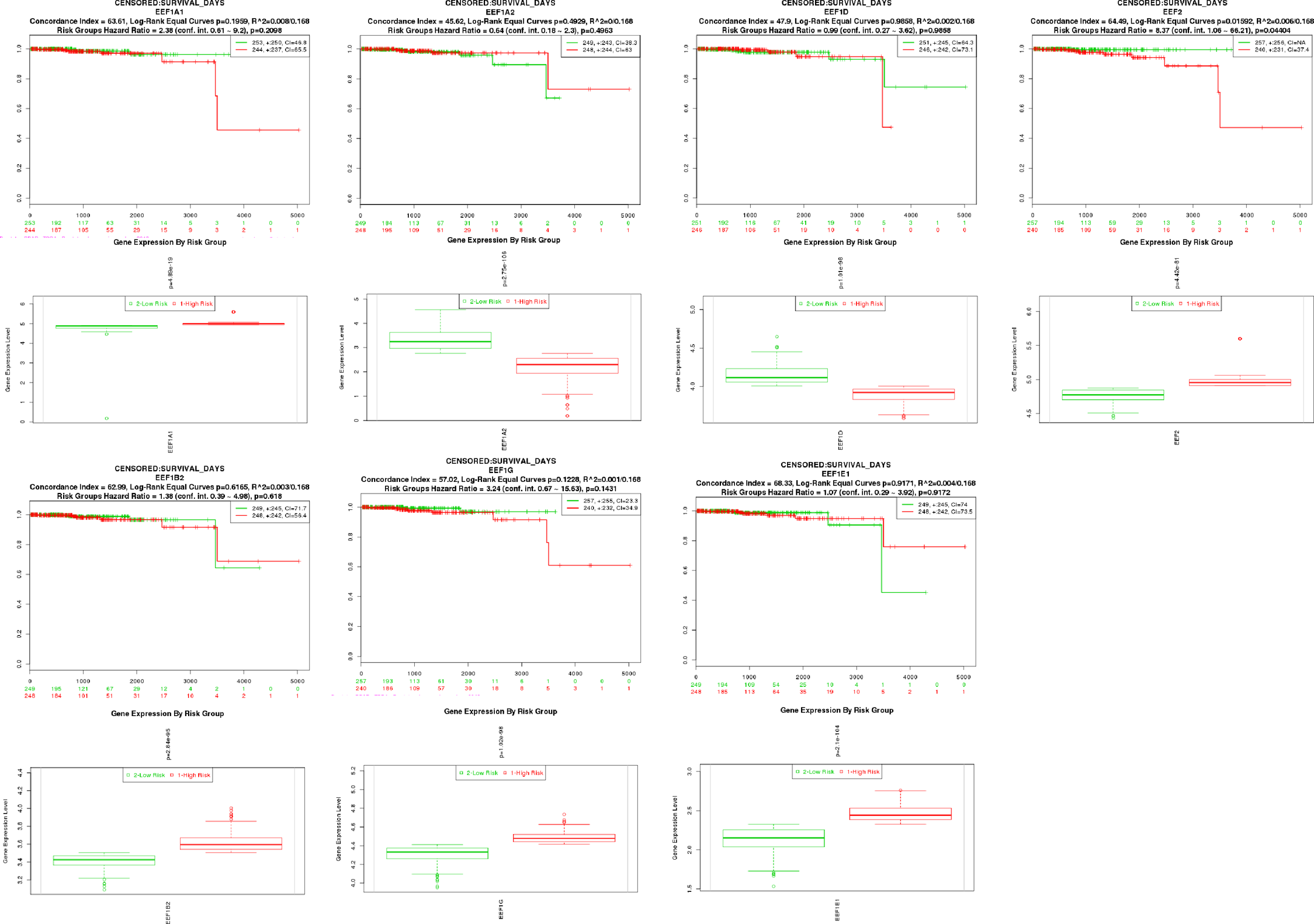

Supplement: S2 Fig — Upper panel shows the Kaplan-Meier curve of risk groups for each gene. The concordance index and p-value of log-rank testing equality of survival curves are indicated. The X-axis represents the time (days) of the study. The number of samples not presenting the event at the matching time has been shown in rows with corresponding color. Lower panel shows the box plots indicating the difference in expression of gene between risk groups, p-values are derived from t-test between both groups. The Y-axis shows the expression levels. In both panels, green color shows low risk group and red color shows high risk group. (TIF) [file pone.0191377.s002.tif]

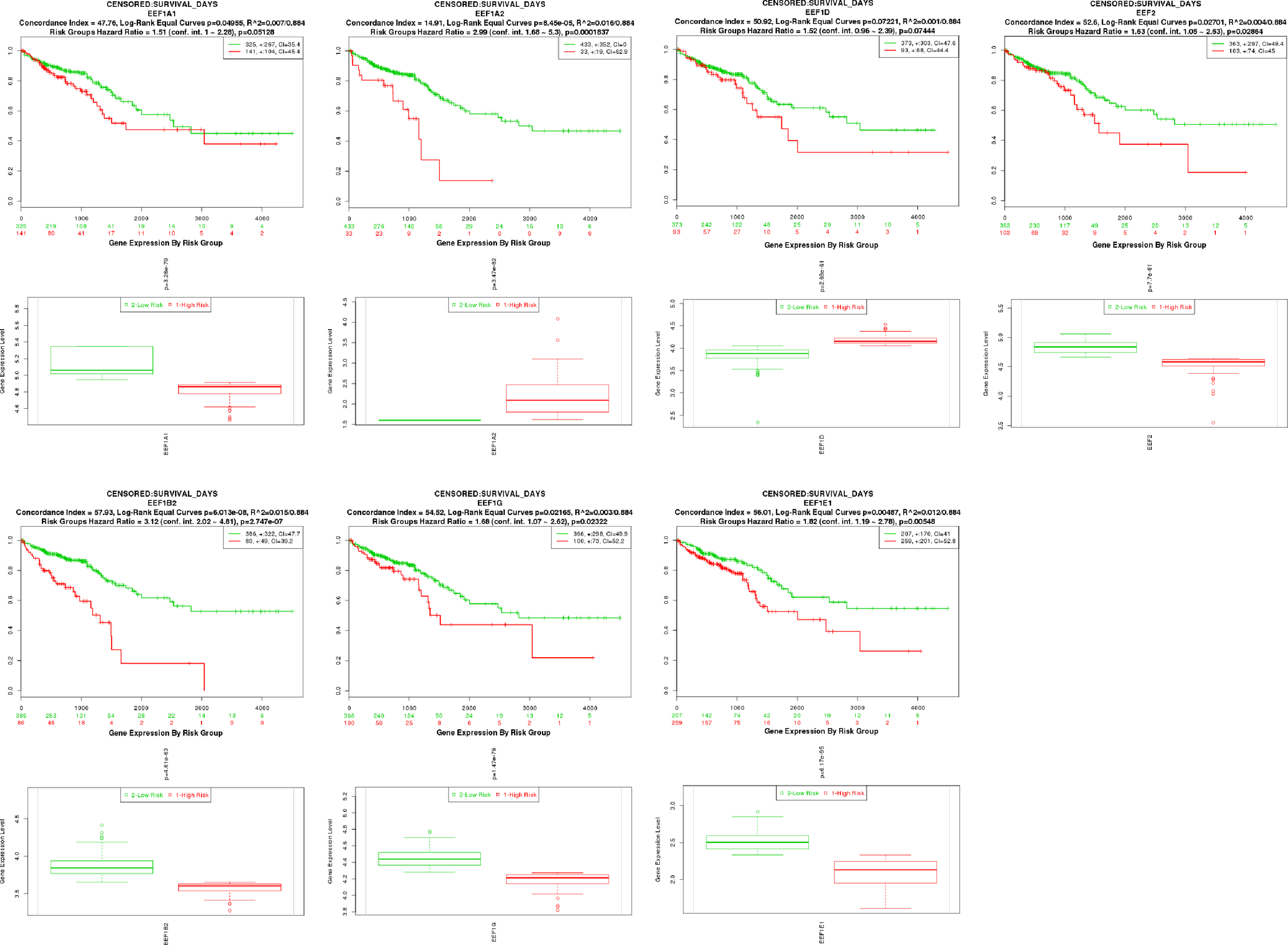

Supplement: S3 Fig — Upper panel shows the Kaplan-Meier curve of risk groups for each gene. The concordance index and p-value of log-rank testing equality of survival curves are indicated. The X-axis represents the time (days) of the study. The number of samples not presenting the event at the matching time has been shown in rows with corresponding color. Lower panel shows the box plots indicating the difference in expression of gene between risk groups, p-values are derived from t-test between both groups. The Y-axis shows the expression levels. In both panels, green color shows low risk group and red color shows high risk group. (TIF) [file pone.0191377.s003.tif]

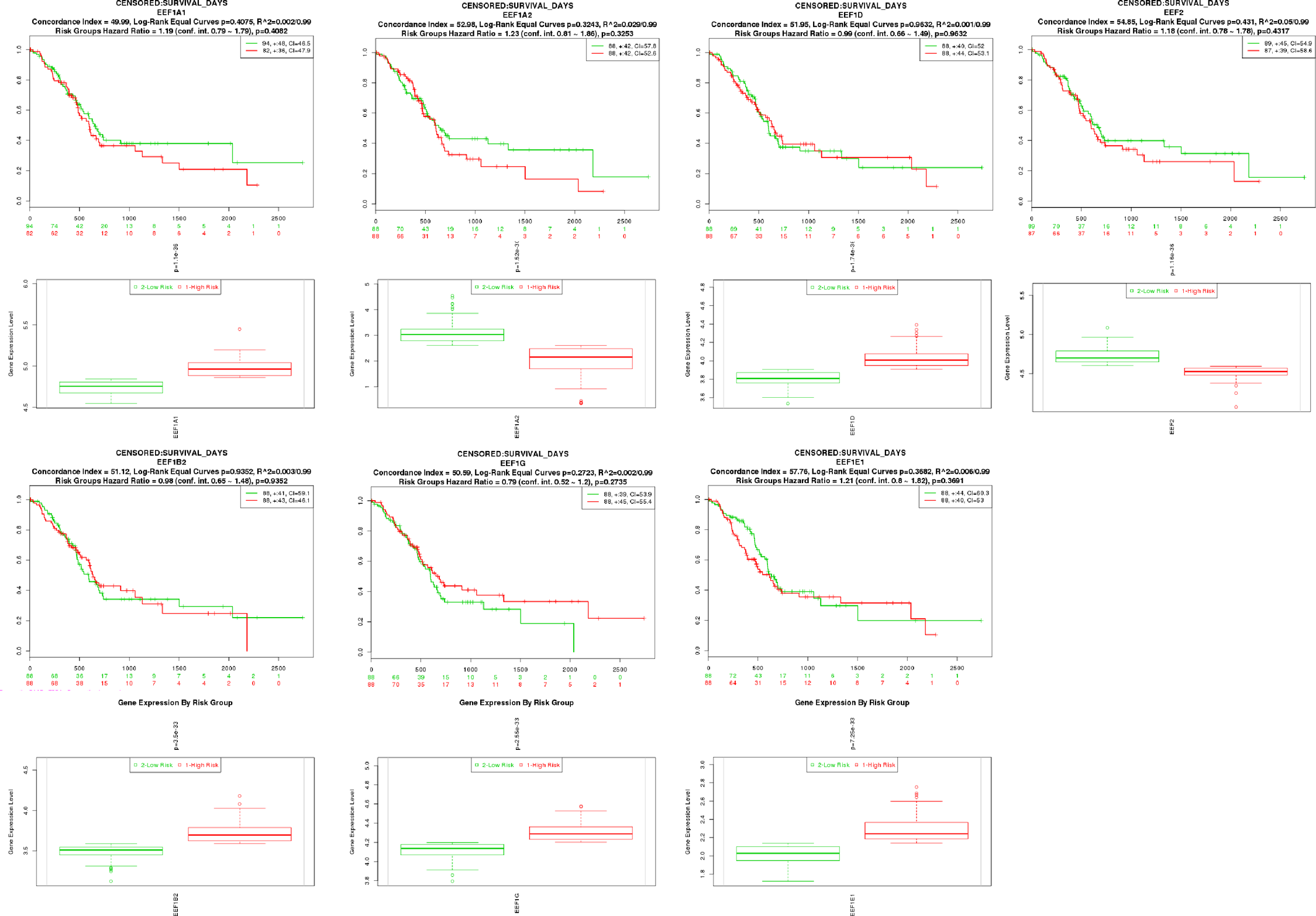

Supplement: S4 Fig — Upper panel shows the Kaplan-Meier curve of risk groups for each gene. The concordance index and p-value of log-rank testing equality of survival curves are indicated. The X-axis represents the time (days) of the study. The number of samples not presenting the event at the matching time has been shown in rows with corresponding color. Lower panel shows the box plots indicating the difference in expression of gene between risk groups, p-values are derived from t-test between both groups. The Y-axis shows the expression levels. In both panels, green color shows low risk group and red color shows high risk group. (TIF) [file pone.0191377.s004.tif]

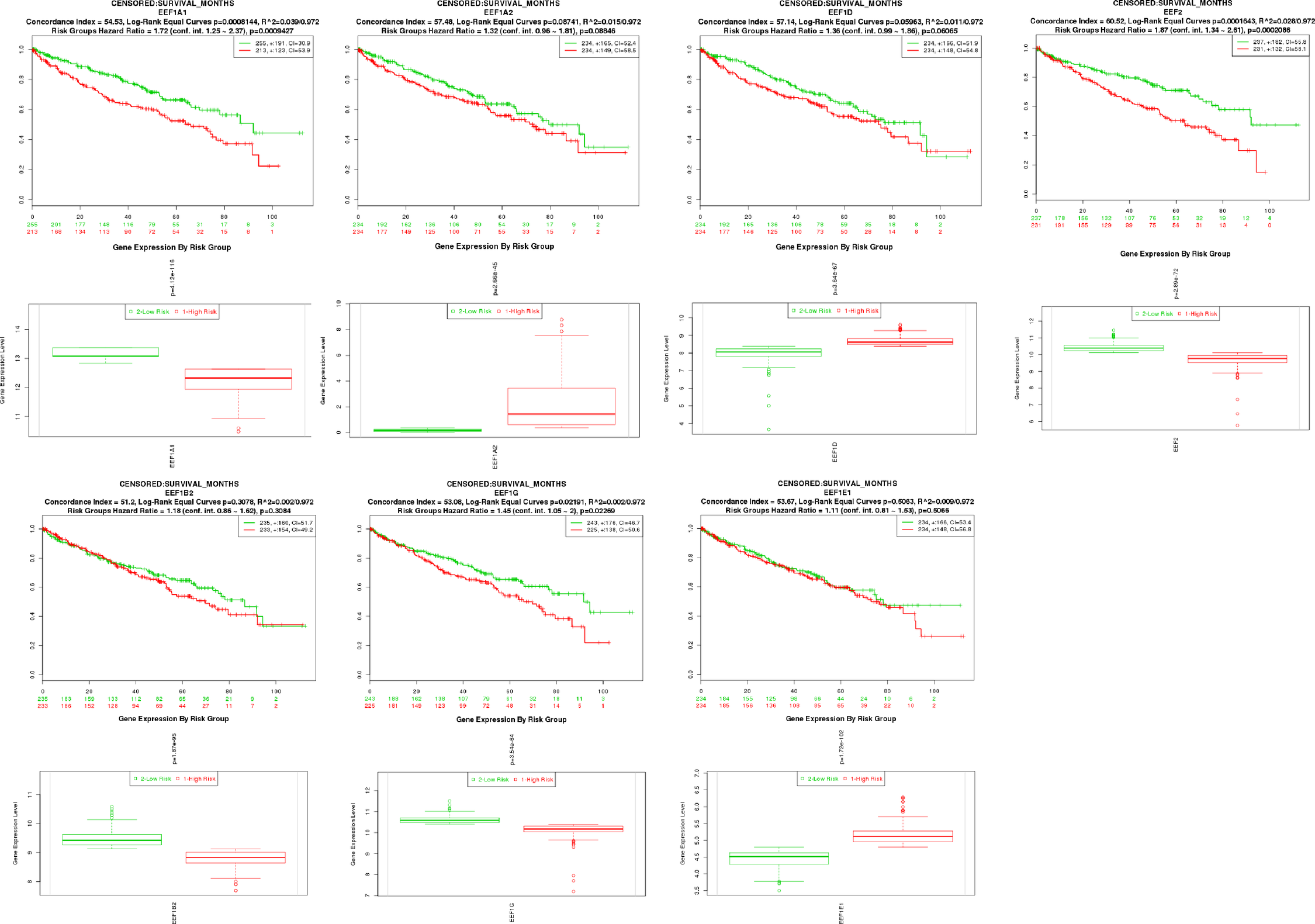

Supplement: S5 Fig — Upper panel shows the Kaplan-Meier curve of risk groups for each gene. The concordance index and p-value of log-rank testing equality of survival curves are indicated. The X-axis represents the time (days) of the study. The number of samples not presenting the event at the matching time has been shown in rows with corresponding color. Lower panel shows the box plots indicating the difference in expression of gene between risk groups, p-values are derived from t-test between both groups. The Y-axis shows the expression levels. In both panels, green color shows low risk group and red color shows high risk group. (TIF) [file pone.0191377.s005.tif]

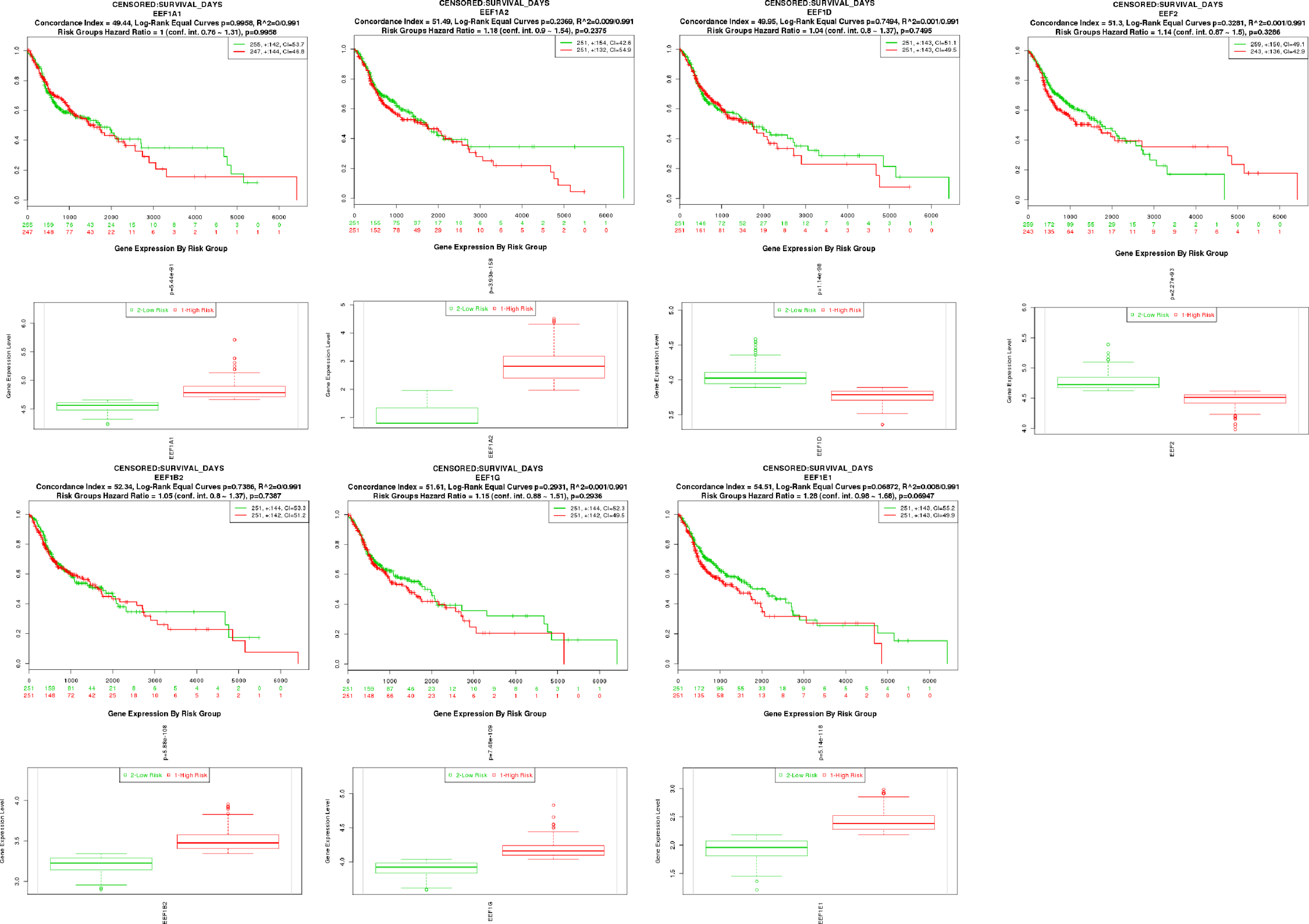

Supplement: S6 Fig — Upper panel shows the Kaplan-Meier curve of risk groups for each gene. The concordance index and p-value of log-rank testing equality of survival curves are indicated. The X-axis represents the time (days) of the study. The number of samples not presenting the event at the matching time has been shown in rows with corresponding color. Lower panel shows the box plots indicating the difference in expression of gene between risk groups, p-values are derived from t-test between both groups. The Y-axis shows the expression levels. In both panels, green color shows low risk group and red color shows high risk group. (TIF) [file pone.0191377.s006.tif]

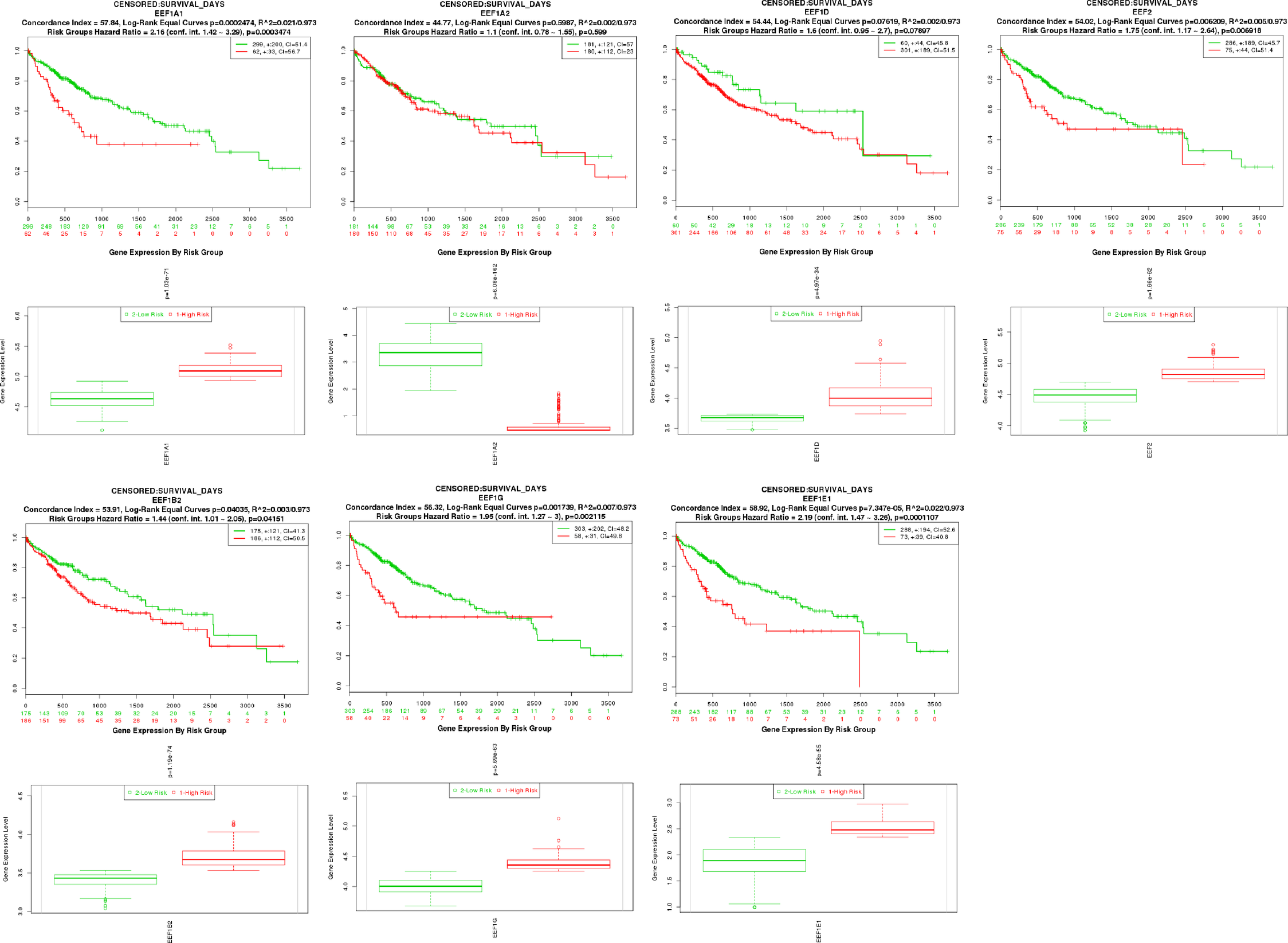

Supplement: S7 Fig — Upper panel shows the Kaplan-Meier curve of risk groups for each gene. The concordance index and p-value of log-rank testing equality of survival curves are indicated. The X-axis represents the time (days) of the study. The number of samples not presenting the event at the matching time has been shown in rows with corresponding color. Lower panel shows the box plots indicating the difference in expression of gene between risk groups, p-values are derived from t-test between both groups. The Y-axis shows the expression levels. In both panels, green color shows low risk group and red color shows high risk group. (TIF) [file pone.0191377.s007.tif]

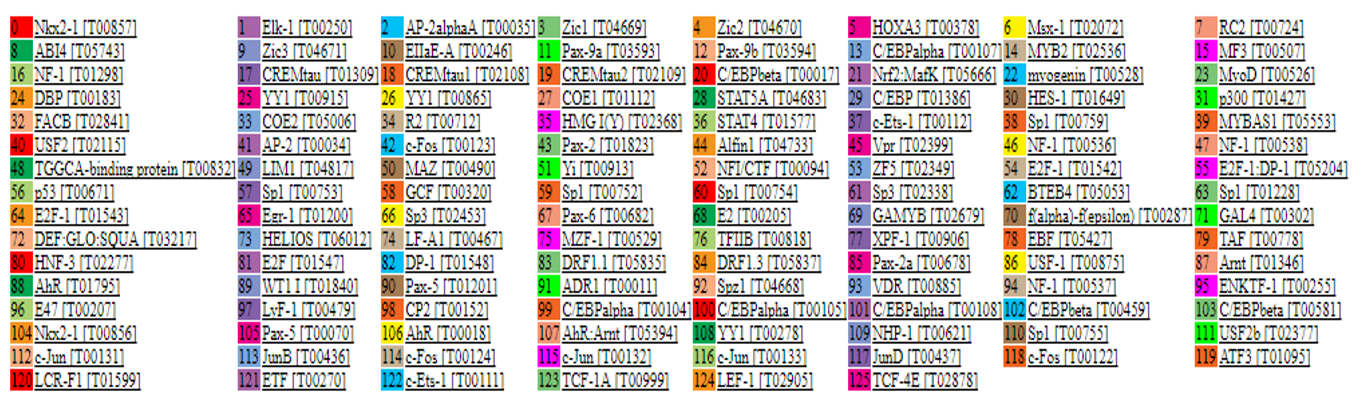

Supplement: S8 Fig — Graphical representation of the common transcription factors binding sites, commonly present in the promoter sequences of all seven, eukaryotic translation elongation factor genes, with a dissimilarity margin less or equal to 15%. Binding site prediction that appear in all seven sequences are shown as boxes of different color and number, square bracket shows TRANSFAC accession ID. (TIF) [file pone.0191377.s008.tif]
