# Supplementary material for: The expression profile and prognostic significance of eukaryotic translation elongation factors in different cancers
Source: PLoS One. 2018 Jan 17;13(1):e0191377. doi: 10.1371/journal.pone.0191377 (PMC5771626; doi:10.1371/journal.pone.0191377)
Supplement: S1 Table — (DOCX) [file pone.0191377.s009.docx]

**Supplementary Table 1. Differential expression analyses of elongation factors in breast cancer**

| **Gene** | **Dataset** | **Normal (Cases)** | **Tumor (Cases)** | **Fold change** | **t-Test** | **p-value** |
| --- | --- | --- | --- | --- | --- | --- |
| EEF1A1 | Radvanyi Breast | Breast(9) | Ductal Breast Carcinoma in Situ (3) | -2.239 | -3.572 | 0.04 |
|  | Richardson Breast | Breast (7) | Ductal Breast Carcinoma (40) | -4.284 | -8.857 | 4.11E-10 |
|  | Karnoub Breast | Breast (15) | Invasive Ductal Breast Carcinoma (7) | -2.481 | -5.793 | 1.18E-4 |
|  | Ma Breast | Breast (14) | Invasive Ductal Breast Carcinoma (9) | -3.083 | -4.585 | 8.46E-5 |
|  | Zhao Breast | Breast (3) | Lobular Breast Carcinoma (20) | -2.022 | -5.056 | 0.005 |
|  |  | Breast (3) | Invasive Ductal Breast Carcinoma (38) | -2.242 | -5.774 | 0.003 |
|  | Finak Breast | Breast (6) | Invasive Breast Carcinoma (53) | -49.866 | -21.109 | 2.62E-18 |
| EEF1A2 | Radvanyi Breast | Breast (7) | Invasive Mixed Breast Carcinoma (3) | 3.033 | 3.684 | 0.003 |
|  | Curtis Breast | Breast (144) | Mucinous Breast Carcinoma (46) | 8.965 | 11.063 | 3.19E-16 |
|  |  | Breast (144) | Invasive Ductal Breast Carcinoma (1,556) | 4.962 | 20.432 | 3.55E-54 |
|  |  | Breast (144) | Invasive Ductal and Invasive Lobular Breast Carcinoma (90) | 4.174 | 10.113 | 7.88E-19 |
|  |  | Breast (144) | Invasive Lobular Breast Carcinoma (148) | 3.584 | 10.455 | 7.00E-22 |
|  |  | Breast (144) | Tubular Breast Carcinoma (67) | 3.513 | 8.897 | 6.81E-15 |
|  | TCGA Breast | Breast (61) | Invasive Ductal Breast Carcinoma (389) | 3.636 | 13.971 | 2.94E-30 |
|  |  | Breast (61) | Intraductal Cribriform Breast Adenocarcinoma (3) | 17.043 | 11.258 | 0.002 |
| EEF1B2 | Ma Breast 4 | Breast (14) | Invasive Ductal Breast Carcinoma (9) | -2.001 | -4.277 | 5.12E-4 |
|  | Finak Breast | Breast (6) | Invasive Breast Carcinoma (53) | -22.465 | -19.569 | 2.29E-24 |
| EEF1G | TCGA Breast | Breast (61) | Intraductal Cribriform Breast Adenocarcinoma (3) | -2.224 | -16.198 | 3.43E-21 |
|  | Ma Breast 4 | Breast (14) | Ductal Breast Carcinoma in Situ (9) | -2.565 | -7.474 | 3.02E-7 |
|  |  | Breast (14) | Invasive Ductal Breast Carcinoma (9) | -2.119 | 5.237 | 5.46E-5 |
|  | Finak Breast | Breast (6) | Invasive Breast Carcinoma (53) | -14.763 | -22.848 | 5.04E-26 |
| EEF1D | Finak Breast | Breast (6) | Invasive Breast Carcinoma (53) | -22.133 | -21.694 | 3.03E-18 |
| EEF2 | Finak Breast | Breast (6) | Invasive Breast Carcinoma (53) | -11.737 | -26.731 | 3.20E-34 |
