# Supplementary material for: The expression profile and prognostic significance of eukaryotic translation elongation factors in different cancers
Source: PLoS One. 2018 Jan 17;13(1):e0191377. doi: 10.1371/journal.pone.0191377 (PMC5771626; doi:10.1371/journal.pone.0191377)
Supplement: S2 Table — Abbreviations: OS—Overall patient survival, FP—First progression, PPS—Post progression survival, DMFS—Distance metastasis free survival, RFS—Relapse free survival. p-values ≤ 0.05 were considered statistically significant and have been denoted in bold. (DOCX) [file pone.0191377.s010.docx]

**Supplementary Table 2**: Kaplan-Meier plotter data showing the correlation between different elongation factors and survival outcomes in breast cancer

| **Gene** | **Dataset/**  **Affymetrix ID** | **Survival outcome** | **No. of Cases** | **HR** | **95% CI** | **p-value** |
| --- | --- | --- | --- | --- | --- | --- |
| EEF1A1 | 227708_at | OS | 626 | 0.9 | 0.66-1.23 | 0.5 |
|  |  | RFS | 1764 | 0.82 | 0.7-0.96 | **0.012** |
|  |  | DMFS | 664 | 0.7 | 0.5-0.97 | **0.029** |
|  |  | PPS | 173 | 1.15 | 0.8-1.64 | 0.46 |
| EEF1A2 | 204540_at | OS | 1402 | 0.97 | 0.79-1.21 | 0.81 |
|  |  | RFS | 3951 | 1.0 | 0.9-1.11 | 0.97 |
|  |  | DMFS | 1746 | 1.19 | 0.98-1.45 | 0.07 |
|  |  | PPS | 414 | 0.92 | 0.72-1.17 | 0.49 |
| EEF1B2 | 200705_s_at | OS | 1402 | 0.78 | 0.63-0.97 | 0.**023** |
|  |  | RFS | 3951 | 1.02 | 0.91-1.14 | 0.74 |
|  |  | DMFS | 1746 | 0.82 | 0.68-1 | **0.05** |
|  |  | PPS | 414 | 0.93 | 0.73-1.19 | 0.59 |
| EEF1G | 211345_x_at | OS | 1402 | 0.83 | 0.67-1.03 | 0.097 |
|  |  | RFS | 3951 | 0.75 | 0.71-0.88 | **1.6e-05** |
|  |  | DMFS | 1746 | 0.71 | 0.59-0.87 | **0.00064** |
|  |  | PPS | 414 | 0.87 | 0.69-1.11 | 0.28 |
| EEF1D | 203113_s_at | OS | 1402 | 0.93 | 0.75-1.15 | 0.52 |
|  |  | RFS | 3951 | 1.23 | 1.1-1.37 | **0.00017** |
|  |  | DMFS | 1746 | 0.88 | 0.72-1.07 | 0.19 |
|  |  | PPS | 414 | 0.9 | 0.7-1.14 | 0.38 |
| EEF1E1 | 204905_s_at | OS | 1402 | 1.23 | 0.99-1.52 | 0.058 |
|  |  | RFS | 3951 | 1.44 | 1.29-1.6 | **6.3e-11** |
|  |  | DMFS | 1746 | 1.23 | 1.01-1.49 | **0.036** |
|  |  | PPS | 414 | 1.22 | 0.95-1.55 | 0.11 |
| EEF2 | 200094_s_at | OS | 1402 | 0.73 | 0.59-0.9 | **0.0032** |
|  |  | RFS | 3951 | 0.8 | 0.72-0.89 | **5.6e-05** |
|  |  | DMFS | 1746 | 0.6 | 0.49-0.73 | **28e-07** |
|  |  | PPS | 414 | 0.98 | 0.76-1.24 | 0.84 |
