# Supplementary material for: The expression profile and prognostic significance of eukaryotic translation elongation factors in different cancers
Source: PLoS One. 2018 Jan 17;13(1):e0191377. doi: 10.1371/journal.pone.0191377 (PMC5771626; doi:10.1371/journal.pone.0191377)
Supplement: S3 Table — Abbreviations: OS: overall survival; RFS: relapse free survival; DMFS: distant metastasis free survival; PPS: post progression survival; HR: Hazard radio; CI: Confidence interval. (DOCX) [file pone.0191377.s011.docx]

| **Gene symbol** | **Survival outcome** | **Basal** | | **Luminal A** | | **Luminal B** | | **HER2+** | |
| --- | --- | --- | --- | --- | --- | --- | --- | --- | --- |
|  |  | **HR (95% CI)** | ***p* value** | **HR (95% CI)** | ***p* value** | **HR (95% CI)** | ***p* value** | **HR (95% CI)** | ***p* value** |
| EEF1A1 | OS | 2.33 | **0.013** | 0.77 | 0.31 | 1.27 | 0.49 | 0.86 | 0.71 |
|  | RFS | 1.14 | 0.44 | 0.81 | 0.088 | 1.24 | 0.17 | 1.01 | 0.97 |
|  | DMFS | 1.42 | 0.33 | 0.78 | 0.37 | 1.01 | 0.99 | 0.87 | 0.71 |
|  | PPS | 2.5 | **0.039** | 1.33 | 0.33 | 0.67 | 0.28 | 1.34 | 0.49 |
| EEF1A2 | OS | 1.6 | 0.065 | 1.27 | 0.18 | 1.29 | 0.18 | 0.59 | 0.11 |
|  | RFS | 1.22 | 0.12 | 1.12 | 0.19 | 1.18 | 0.094 | 1.11 | 0.59 |
|  | DMFS | 1.61 | 0.069 | 1.54 | **0.0034** | 1.35 | 0.095 | 0.96 | 0.9 |
|  | PPS | 1.39 | 0.27 | 1.14 | 0.51 | 0.92 | 0.7 | 0.31 | **0.0029** |
| EEF1B2 | OS | 1.5 | 0.11 | 0.53 | **0.00048** | 0.64 | **0.019** | 0.82 | 0.56 |
|  | RFS | 1.29 | 0.051 | 0.89 | 0.17 | 1.13 | 0.2 | 0.89 | 0.55 |
|  | DMFS | 1.69 | **0.046** | 0.7 | **0.015** | 0.84 | 0.32 | 0.7 | 0.25 |
|  | PPS | 1.58 | 0.12 | 0.69 | 0.068 | 0.78 | 0.25 | 1.53 | 0.26 |
| EEF1G | OS | 1.63 | 0.055 | 0.63 | **0.013** | 0.77 | 0.17 | 0.77 | 0.42 |
|  | RFS | 0.94 | 0.62 | 0.76 | **0.0014** | 0.84 | 0.077 | 0.68 | **0.05** |
|  | DMFS | 1.36 | 0.24 | 0.7 | **0.015** | 0.8 | 0.22 | 0.87 | 0.71 |
|  | PPS | 1.52 | 0.16 | 0.59 | **0.009** | 1.13 | 0.57 | 0.87 | 0.71 |
| EEF1D | OS | 1.54 | **0.087** | 1.04 | 0.84 | 0.77 | 0.22 | 1.04 | 0.91 |
|  | RFS | 1.68 | **5.8e-05** | 1.26 | **0.0089** |  |  | 1.26 | 0.24 |
|  | DMFS | 1.32 | 0.28 | 0.89 | 0.43 |  |  | 0.79 | 0.46 |
|  | PPS | 1.10 | 0.56 | 1.02 | 0.9 |  |  | 0.91 | 0.82 |
| EEF1E1 | OS | 0.91 | 0.72 | 1.21 | 0.29 | 1.01 | 0.94 | 0.61 | 0.14 |
|  | RFS | 1.05 | 0.72 | 1.45 | **2.1e-05** | 1.51 | **2.9e-05** | 0.8 | 0.25 |
|  | DMFS | 0.95 | 0.84 | 1.11 | 0.47 | 1.41 | 0.057 | 0.42 | **0.0082** |
|  | PPS | 2 | **0.02** | 1.2 | 0.36 | 0.78 | 0.26 | 1.3 | 0.49 |
| EEF2 | OS | 1.42 | 0.17 | 0.74 | 0.089 | 0.62 | **0.01** | 1.09 | 0.79 |
|  | RFS | 1.43 | **0.0055** | 0.79 | **0.0076** | 0.88 | 0.21 | 1.21 | 0.32 |
|  | DMFS | 1.39 | 0.21 | 0.59 | **3e-04** | 0.68 | **0.035** | 1.36 | 0.33 |
|  | PPS | 2.4 | **0.0034** | 0.97 | 0.9 | 1.06 | 0.8 | 1.21 | 0.62 |
|  | | | | | | | | | |

**Supplementary Table 3: The correlation between elongation factors and survival outcomes in breast cancer patients restricted by intrinsic subtypes**
