# Supplementary material for: The expression profile and prognostic significance of eukaryotic translation elongation factors in different cancers
Source: PLoS One. 2018 Jan 17;13(1):e0191377. doi: 10.1371/journal.pone.0191377 (PMC5771626; doi:10.1371/journal.pone.0191377)
Supplement: S4 Table — (DOCX) [file pone.0191377.s012.docx]

**Supplementary Table 4: Differential expression analyses of elongation factors in lung cancer**

| **Gene** | **Dataset** | **Normal (Cases)** | **Tumor (Cases)** | **Fold change** | **t-Test** | **p-value** |
| --- | --- | --- | --- | --- | --- | --- |
| EEF1A1 | Garber Lung | Lung (5) | Small Cell Lung Carcinoma (4) | -2.640 | -3.604 | 0.004 |
|  |  | Lung (5) | Lung Adenocarcinoma (40) | -2.496 | -4.139 | 0.002 |
|  | Hou Lung | Lung (65) | Lung Adenocarcinoma (45) | -2.133 | -7.584 | 1.58E-10 |
|  |  | Lung (65) | Squamous Cell Lung Carcinoma (27) | -2.819 | -9.169 | 8.48E-11 |
| EEF1A2 | Bhattacharjee Lung | Lung (17) | Lung Adenocarcinoma (132) | 9.835 | 5.674 | 5.90E-6 |
|  |  | Lung (17) | Lung Carcinoid Tumor (20) | 46.009 | 9.286 | 1.79E-9 |
|  |  | Lung (17) | Small Cell Lung Carcinoma (6) | 18.747 | 4.026 | 0.002 |
|  |  | Lung (17) | Squamous Cell Lung Carcinoma (21) | 6.541 | 3.287 | 0.001 |
|  | Stearman Lung | Lung (19) | Lung Adenocarcinoma (20) | 20.160 | 6.698 | 1.23E-7 |
|  | Beer Lung | Lung (10) | Lung Adenocarcinoma (86) | 2.996 | 6.437 | 5.10E-6 |
|  | Su Lung | Lung (30) | Lung Adenocarcinoma (27) | 7.006 | 5.964 | 3.17E-7 |
|  | Selamat Lung | Lung (58) | Lung Adenocarcinoma (58) | 7.458 | 8.955 | 8.10E-13 |
| EEF1D | Bhattacharjee Lung | Lung (17) | Lung Carcinoid Tumor (20) | -7.719 | -6.498 | 8.06E-7 |
| EEF1E1 | Bhattacharjee Lung | Lung (17) | Small Cell Lung Carcinoma (6) | 3.010 | 3.991 | 3.32E-4 |
|  |  | Lung (17) | Lung Carcinoid Tumor (20) | 2.271 | 3.662 | 8.20E-4 |
|  |  | Lung (17) | Squamous Cell Lung Carcinoma (21) | 2.868 | 3.030 | 0.002 |
|  | Hou Lung | Lung (65) | Large Cell Lung Carcinoma (19) | 2.438 | 6.138 | 2.75E-6 |
