# Supplementary material for: The expression profile and prognostic significance of eukaryotic translation elongation factors in different cancers
Source: PLoS One. 2018 Jan 17;13(1):e0191377. doi: 10.1371/journal.pone.0191377 (PMC5771626; doi:10.1371/journal.pone.0191377)
Supplement: S5 Table — Abbreviations: OS: overall survival; RFS: relapse free survival; DMFS: distant metastasis free survival; PPS: post progression survival; HR: Hazard radio; CI: Confidence interval. (DOCX) [file pone.0191377.s013.docx]

**Supplementary Table 5: Kaplan-Meier plotter data showing the correlation between different elongation factors and survival outcomes in lung cancer**

| **Gene** | **Dataset/**  **Affymetrix ID** | **Survival outcome** | **No. of Cases** | **HR** | **95% CI** | **p-value** |
| --- | --- | --- | --- | --- | --- | --- |
| EEF1A1 | 227708_at | OS | 1145 | 0.58 | 0.49-0.69 | **1.9e-10** |
|  |  | FP | 596 | 0.7 | 0.53-0.92 | **0.0095** |
|  |  | PPS | 138 | 0.7 | 0.46-1.08 | 0.11 |
| EEF1A2 | 204540_at | OS | 1926 | 1.2 | 1.05-1.36 | **0.0054** |
|  |  | FP | 982 | 1.76 | 1.45-2.14 | **8.3e-09** |
|  |  | PPS | 344 | 1.08 | 0.84-1.4 | 0.53 |
| EEF1B2 | 200705_s_at | OS | 1926 | 1.21 | 1.07-1.38 | **0.0031** |
|  |  | FP | 982 | 1.11 | 0.92-1.34 | 0.29 |
|  |  | PPS | 344 | 1.11 | 0.87-1.44 | 0.4 |
| EEF1G | 211345_x_at | OS | 1926 | 1.34 | 1.18-1.52 | **7e-06** |
|  |  | FP | 982 | 1.43 | 1.18-1.74 | **0.00024** |
|  |  | PPS | 344 | 1.11 | 0.86-1.43 | 0.43 |
| EEF1D | 203113_s_at | OS | 1926 | 1.07 | 0.95-1.22 | 0.27 |
|  |  | FP | 982 | 0.9 | 0.75-1.1 | 0.3 |
|  |  | PPS | 344 | 1.12 | 0.87-1.44 | 0.39 |
| EEF1E1 | 204905_s_at | OS | 1926 | 1.32 | 1.1-1.58 | **0.0033** |
|  |  | FP | 982 | 1.6 | 1.32-1.94 | **1.76e-06** |
|  |  | PPS | 344 | 0.91 | 0.71-1.17 | 0.47 |
| EEF2 | 200094_s_at | OS | 1926 | 1.1 | 0.97-1.25 | 0.13 |
|  |  | FP | 982 | 0.94 | 0.78-1.14 | 0.52 |
|  |  | PPS | 344 | 1.07 | 0.83-1.38 | 0.59 |
