# Supplementary material for: The expression profile and prognostic significance of eukaryotic translation elongation factors in different cancers
Source: PLoS One. 2018 Jan 17;13(1):e0191377. doi: 10.1371/journal.pone.0191377 (PMC5771626; doi:10.1371/journal.pone.0191377)
Supplement: S6 Table — Abbreviations: OS: overall survival; RFS: relapse free survival; DMFS: distant metastasis free survival; PPS: post progression survival; HR: Hazard radio; CI: Confidence interval. p-values ≤ 0.05 were considered statistically significant and have been denoted in bold. (DOCX) [file pone.0191377.s014.docx]

**Supplementary Table 6: The Correlation between Elongation factors and survival outcomes in lung cancer patients stratified by histological types and tumor stages**

| **Gene symbol** | **Survival outcome** | **Histology** | | | | **Tumor stage** | | | | | |
| --- | --- | --- | --- | --- | --- | --- | --- | --- | --- | --- | --- |
|  |  | **Adenocarcinoma** | | **Squamous cell carcinoma** | | **1** | | **2** | | | |
|  |  | **HR (95% CI)** | ***p* value** | **HR (95% CI)** | ***p* value** | **HR (95% CI)** | ***p* value** | **HR (95% CI)** | | ***p* value** | |
| EEF1A1 | OS | 0.44 | **4.7e-11** | 1.03 | 0.86 | 0.42 | **2.6e-07** | 0.72 | 0.15 | |  |
|  | FP | 0.47 | **4.9e-06** | 1.56 | 0.091 | 0.53 | **0.0053** | 1.07 | 0.82 | |  |
|  | PPS | 0.71 | 0.17 | 1.25 | 0.67 | 0.54 | **0.044** | 0.63 | 0.19 | |  |
| EEF1A2 | OS | 1.63 | **4e-05** | 1.13 | 0.31 | 1.78 | **3e-05** | 1.43 | 0.058 | |  |
|  | FP | 1.64 | **0.0019** | 1.18 | 0.54 | 1.26 | 0.3 | 1.3 | 0.32 | |  |
|  | PPS | 1 | 0.99 | 1.03 | 0.95 | 1.09 | 0.78 | 1.44 | 0.27 | |  |
| EEF1B2 | OS | 1.39 | **0.0079** | 1.09 | 0.47 | 0.93 | 0.75 | 1.01 | 0.95 | |  |
|  | FP | 1.14 | 0.41 | 0.72 | 0.21 | 1.53 | **0.0026** | 0.58 | 0.041 | |  |
|  | PPS | 0.95 | 0.83 | 0.85 | 0.76 | 0.88 | 0.68 | 1.71 | 0.1 | |  |
| EEF1G | OS | 1.85 | **2.9e-07** | 1.2 | 0.14 | 1.97 | **9.2e-07** | 1.94 | **0.00034** | |  |
|  | FP | 1.64 | **0.0018** | 0.77 | 0.32 | 1.57 | **0.043** | 0.74 | 0.25 | |  |
|  | PPS | 1.27 | 0.32 | 0.85 | 0.76 | 1.52 | 0.17 | 1.47 | 0.24 | |  |
| EEF1D | OS | 1.26 | 0.055 | 1.06 | 0.61 | 1.43 | **0.0099** | 1.59 | 0.16 | |  |
|  | FP | 0.9 | 0.5 | 0.56 | 0.03 | 1.01 | 0.95 | 1.05 | 0.84 | |  |
|  | PPS | 1.18 | 0.48 | 0.85 | 0.76 | 1.54 | 0.16 | 1.59 | 0.16 | |  |
| EEF1E1 | OS | 1.25 | 0.063 | 1.28 | **0.044** | 1.55 | **0.0019** | 1.09 | 0.64 | |  |
|  | FP | 1.14 | 0.41 | 1.59 | 0.079 | 0.85 | 0.48 | 1.3 | 0.31 | |  |
|  | PPS | 0.64 | 0.064 | 1.1 | 0.85 | 0.63 | 0.13 | 0.66 | 0.21 | |  |
| EEF2 | OS | 1.5 | **0.00073** | 0.92 | 0.49 | 1.72 | **9.8e-05** | 1.07 | 0.7 | |  |
|  | FP | 1.01 | 0.93 | 0.65 | 0.099 | 0.95 | 0.82 | 0.74 | 0.26 | |  |
|  | PPS | 1.4 | 0.18 | 0.85 | 0.76 | 2.31 | **0.0061** | 1.38 | 0.32 | |  |
