# Supplementary material for: The expression profile and prognostic significance of eukaryotic translation elongation factors in different cancers
Source: PLoS One. 2018 Jan 17;13(1):e0191377. doi: 10.1371/journal.pone.0191377 (PMC5771626; doi:10.1371/journal.pone.0191377)
Supplement: S7 Table — (DOCX) [file pone.0191377.s015.docx]

**Supplementary Table 7: Differential expression analyses of elongation factors in gastric cancer**

| **Gene** | **Dataset** | **Normal (Cases)** | **Tumor (Cases)** | **Fold change** | **t-Test** | **p-value** |
| --- | --- | --- | --- | --- | --- | --- |
| EEF1A2 | Cho Gastric | Gastric Tissue (19) | Gastric Adenocarcinoma (4) | -8.182 | -10.131 | 8.38E-10 |
|  | Cho Gastric | Gastric Tissue (19) | Gastric Mixed Adenocarcinoma (10) | -7.309 | -8.111 | 5.32E-9 |
|  | Cho Gastric | Gastric Tissue (19) | Gastric Intestinal Type Adenocarcinoma (20) | -6.984 | -8.530 | 9.08E-10 |
|  | Cho Gastric | Gastric Tissue (19) | Diffuse Gastric Adenocarcinoma (31) | -6.397 | -7.469 | 2.77E-9 |
|  | DErrico Gastric | Gastric Mucosa (31) | Gastric Mixed Adenocarcinoma (4) | -3.540 | -7.503 | 1.73E-4 |
|  | DErrico Gastric | Gastric Mucosa (31) | Diffuse Gastric Adenocarcinoma (6) | -2.213 | -3.215 | 0.008 |
