# Supplementary material for: The expression profile and prognostic significance of eukaryotic translation elongation factors in different cancers
Source: PLoS One. 2018 Jan 17;13(1):e0191377. doi: 10.1371/journal.pone.0191377 (PMC5771626; doi:10.1371/journal.pone.0191377)
Supplement: S8 Table — Abbreviations: OS: overall survival; RFS: relapse free survival; DMFS: distant metastasis free survival; PPS: post progression survival; HR: Hazard radio; CI: Confidence interval. p-values ≤ 0.05 were considered statistically significant and have been denoted in bold. (DOCX) [file pone.0191377.s016.docx]

**Supplementary Table 8: Kaplan-Meier plotter data showing the correlation between different elongation factors and survival outcomes in gastric cancer**

| **Gene** | **Dataset/**  **Affymetrix ID** | **Survival outcome** | **No. of Cases** | **HR** | **95% CI** | **p-value** |
| --- | --- | --- | --- | --- | --- | --- |
| EEF1A1 | 227708_at | OS | 631 | 0.82 | 0.66-1.02 | 0.074 |
|  |  | FP | 522 | 0.8 | 0.61-1.03 | 0.087 |
| EEF1A2 | 204540_at | OS | 876 | 1.41 | 1.18-1.69 | **2e-04** |
|  |  | FP | 641 | 1.35 | 1.09-1.66 | **0.0057** |
| EEF1B2 | 200705_s_at | OS | 876 | 0.63 | 0.53-0.74 | **8e-08** |
|  |  | FP | 641 | 0.6 | 0.49-0.73 | **3.8e-07** |
| EEF1G | 211345_x_at | OS | 846 | 1.53 | 1.28-1.83 | **2.6e-06** |
|  |  | FP | 641 | 1.44 | 1.16-1.8 | **0.00095** |
| EEF1D | 203113_s_at | OS | 876 | 1.14 | 0.94-1.38 | 0.18 |
|  |  | FP | 641 | 1.21 | 0.98-1.49 | 0.072 |
| EEF1E1 | 204905_s_at | OS | 876 | 0.67 | 0.55-0.82 | **7.6e-05** |
|  |  | FP | 641 | 0.6 | 0.46-0.77 | **8.6e-05** |
| EEF2 | 200094_s_at | OS | 876 | 1.08 | 0.91-1.29 | 0.38 |
|  |  | FP | 641 | 0.88 | 0.71-1.07 | 0.2 |
