# Supplementary material for: The expression profile and prognostic significance of eukaryotic translation elongation factors in different cancers
Source: PLoS One. 2018 Jan 17;13(1):e0191377. doi: 10.1371/journal.pone.0191377 (PMC5771626; doi:10.1371/journal.pone.0191377)
Supplement: S9 Table — Abbreviations: OS: overall survival; RFS: relapse free survival; DMFS: distant metastasis free survival; PPS: post progression survival; HR: Hazard radio; CI: Confidence interval. p-values ≤ 0.05 were considered statistically significant and have been denoted in bold. (DOCX) [file pone.0191377.s017.docx]

**Supplementary Table 9: The correlation between elongation factors and survival outcomes in gastric cancer patients restricted by tumor stages and Her2 status**

| **Gene symbol** | **Survival outcome** | **Tumor stage** | | | | | | | | **HER2 status** | | | | |  |
| --- | --- | --- | --- | --- | --- | --- | --- | --- | --- | --- | --- | --- | --- | --- | --- |
|  |  | **1** | | **2** | | **3** | | **4** | | **Positive** | | **Negative** | | |  |
|  |  | **HR (95% CI)** | ***p* value** | **HR (95% CI)** | ***p* value** | **HR (95% CI)** | ***p* value** | **HR (95% CI)** | ***p* value** | **HR (95% CI)** | ***p* value** | **HR (95% CI)** | | ***p* value** | |
| EEF1A1 | OS | 0.49 | 0.2 | 1.37 | 0.34 | 0.67 | **0.033** | 1.41 | 0.1 | 0.73 | 0.12 | 0.83 | 0.22 | |  |
|  | FP | 0.54 | 0.26 | 0.71 | 0.27 | 0.64 | **0.023** | 0.88 | 0.52 | 0.56 | **0.0098** | 1.16 | 0.33 | |  |
| EEF1A2 | OS | 2.08 | 0.15 | 2.14 | **0.011** | 1.83 | **5.7e-05** | 0.73 | 0.14 | 1.4 | **0.016** | 1.45 | **0.0011** | |  |
|  | FP | 1.49 | 0.47 | 2.59 | **0.0013** | 1.18 | 0.37 | 0.7 | 0.11 | 1.39 | 0.062 | 1.4 | **0.014** | |  |
| EEF1B2 | OS | 0.33 | **0.031** | 0.31 | **0.0097** | 0.6 | **0.00035** | 0.56 | **0.005** | 0.6 | 0.00011 | 0.66 | **0.00028** | |  |
|  | FP | 0.45 | 0.16 | 0.53 | 0.1 | 0.58 | **0.0031** | 0.62 | **0.02** | 0.54 | 0.00017 | 0.64 | **0.00073** | |  |
| EEF1G | OS | 4.11 | **0.0027** | 1.57 | 0.17 | 1.8 | **4.3e-05** | 1.48 | **0.05** | 0.81 | 0.16 | 1.74 | **3.7e-06** | |  |
|  | FP | 2.58 | 0.085 | 1.44 | 0.25 | 1.38 | 0.09 | 1.44 | 0.068 | 0.75 | 0.1 | 1.64 | **0.00019** | |  |
| EEF1D | OS | 3.49 | **0.0086** | 0.61 | 0.1 | 1.19 | 0.25 | 0.78 | 0.28 | 0.78 | 0.059 | 1.34 | **0.015** | |  |
|  | FP | 6.16 | **0.00061** | 0.61 | 0.1 | 1.29 | 0.18 | 1.27 | 0.23 | 0.72 | 0.072 | 1.42 | **0.0083** | |  |
| EEF1E1 | OS | 0.38 | 0.068 | 0.59 | 0.094 | 0.64 | **0.0048** | 0.62 | **0.018** | 1.15 | 0.29 | 0.6 | **4.7e-05** | |  |
|  | FP | 0.49 | 0.22 | 0.63 | 0.14 | 0.6 | **0.013** | 0.69 | 0.11 | 0.87 | 0.43 | 0.56 | **0.00029** | |  |
| EEF2 | OS | 2.1 | 0.15 | 2.14 | 0.058 | 1.57 | **0.0021** | 1.29 | 0.27 | 0.72 | **0.013** | 1.29 | **0.025** | |  |
|  | FP | 1.87 | 0.26 | 2.12 | 0.063 | 1.7 | **0.0058** | 0.87 | **0.5** | 0.59 | 0.0017 | 1.22 | **0.16** | |  |
|  | | | | | | | | | | | | | | |  |
