# Supplementary material for: The expression profile and prognostic significance of eukaryotic translation elongation factors in different cancers
Source: PLoS One. 2018 Jan 17;13(1):e0191377. doi: 10.1371/journal.pone.0191377 (PMC5771626; doi:10.1371/journal.pone.0191377)
Supplement: S10 Table — (DOCX) [file pone.0191377.s018.docx]

**Supplementary Table 10: Differential expression analyses of elongation factors in brain and CNS cancer**

| **Gene** | **Dataset** | **Normal (Cases)** | **Tumor (Cases)** | **Fold change** | **t-Test** | **p-value** |
| --- | --- | --- | --- | --- | --- | --- |
| EEF1A1 | Shai Brain | White Matter (7) | Oligodendroglioma (3) | 2.811 | 7.565 | 8.30E-5 |
|  | Shai Brain | White Matter (7) | Astrocytoma (5) | 2.014 | 4.173 | 9.60E-4 |
|  | Bredel Brain 2 | Brain (4) | Anaplastic Oligodendroglioma (3) | 2.852 | 8.574 | 0.002 |
|  | Bredel Brain 2 | Brain (4) | Oligodendroglioma (5) | 2.369 | 4.679 | 0.003 |
| EEF1A2 | Pomeroy Brain | Cerebellum (4) | Malignant Glioma, NOS (10) | -8.224 | -7.162 | 6.67E-6 |
|  |  | Cerebellum (4) | Classic Medulloblastoma (46) | -5.788 | -6.926 | 3.19E-6 |
|  |  | Cerebellum (4)) | Desmoplastic Medulloblastoma (14) | -19.564 | 4.016 | 6.16E-4 |
|  | TCGA Brain | Brain (10) | Brain Glioblastoma (542) | -11.305 | -39.849 | 1.98E-28 |
|  | Sun Brain | Brain (23) | Glioblastoma (81) | -5.406 | -9.119 | 2.79E-13 |
| EEF1B2 | Pomeroy Brain | Cerebellum (4) | Atypical Teratoid/Rhabdoid Tumor (5) | 2.732 | 3.148 | 0.009 |
|  |  | White Matter (7) | Oligodendroglioma (3) | 3.566 | 4.977 | 6.55E-4 |
| EEF1G | Pomeroy Brain | Cerebellum (4) | Desmoplastic Medulloblastoma (14) | 2.911 | 6.358 | 8.60E-6 |
|  |  | Cerebellum (4) | Atypical Teratoid/Rhabdoid Tumor (5) | 4.146 | 5.530 | 0.001 |
|  |  | Cerebellum (4) | Classic Medulloblastoma (46) | 2.503 | 7.088 | 2.51E-5 |
|  | Rickman Brain | Temporal Lobe (6) | Astrocytoma (45) | 2.123 | 12.373 | 1.08E-6 |
|  | French Brain | Brain (6) | Anaplastic Oligodendroglioma (23) | 2.190 | 8.951 | 8.80E-7 |
| EEF1D | TCGA Brain | Brain (10) | Glioblastoma (5) | 2.030 | 8.660 | 6.43E-5 |
|  |  | Brain (10) | Brain Glioblastoma (542) | 2.504 | 23.716 | 1.18E-11 |
|  | Rickman Brain | Temporal Lobe (6)) | Astrocytoma (45) | 2.653 | 4.850 | 0.002 |
| EEF1E1 | Shai Brain | Brain (14) | Astrocytoma (7) | 2.072 | 7.339 | 1.84E-5 |
|  |  | White Matter (7) | Oligodendroglioma (3) | 4.336 | 8.497 | 0.001 |
| EEF2 | Pomeroy Brain | Cerebellum (4) | Desmoplastic Medulloblastoma (14) | 2.304 | 5.998 | 1.02E-5 |
|  |  | Cerebellum (4) | Classic Medulloblastoma (46) | 2.549 | 10.210 | 1.21E-10 |
|  |  | Cerebellum (4) | Atypical Teratoid/Rhabdoid Tumor (5) | 2.675 | 4.469 | 0.004 |
