# Supplementary material for: The expression profile and prognostic significance of eukaryotic translation elongation factors in different cancers
Source: PLoS One. 2018 Jan 17;13(1):e0191377. doi: 10.1371/journal.pone.0191377 (PMC5771626; doi:10.1371/journal.pone.0191377)
Supplement: S11 Table — (DOCX) [file pone.0191377.s019.docx]

**Supplementary Table 11: Differential expression analyses of elongation factors in prostate cancer.**

| **Gene** | **Dataset** | **Normal (Cases)** | **Tumor (Cases)** | **Fold change** | **t-Test** | **p-value** |
| --- | --- | --- | --- | --- | --- | --- |
| EEF1A2 | Magee Prostate | Prostate Gland (4) | Prostate Carcinoma (8) | 3.649 | 2.984 | 0.007 |
|  | LaTulippe Prostate | Prostate Gland (3) | Prostate Carcinoma (23) | 3.117 | 3.806 | 0.002 |
| EEF1G | Singh Prostate | Prostate Gland (50) | Prostate Carcinoma (52) | 2.978 | 4.112 | 5.30E-5 |
| EEF1D | Singh Prostate | Prostate Gland (50) | Prostate Carcinoma (52) | 3.495 | 3.515 | 3.76E-4 |
| EEF1E1 | Tomlins Prostate | Prostate Gland (20) | Prostate Carcinoma (27) | 2.286 | 4.727 | 1.13E-5 |
|  | Singh Prostate | Prostate Gland (50) | Prostate Carcinoma (52) | 2.508 | 3.745 | 1.52E-4 |
| EEF2 | Singh Prostate | Prostate Gland (50) | Prostate Carcinoma (52) | 2.303 | 5.559 | 1.32E-7 |
