# Supplementary material for: The expression profile and prognostic significance of eukaryotic translation elongation factors in different cancers
Source: PLoS One. 2018 Jan 17;13(1):e0191377. doi: 10.1371/journal.pone.0191377 (PMC5771626; doi:10.1371/journal.pone.0191377)
Supplement: S12 Table — (DOCX) [file pone.0191377.s020.docx]

**Supplementary Table 12: Differential expression analyses of elongation factors in colorectal cancer**

| **Gene** | **Dataset** | **Normal (Cases)** | **Tumor (Cases)** | **Fold change** | **t-Test** | **p-value** |
| --- | --- | --- | --- | --- | --- | --- |
| EEF1D | Hong Colorectal | Colon (12) | Colorectal Carcinoma (70) | 2.405 | 12.508 | 3.89E-14 |
| EEF1E1 | Skrzypczak Colorectal | Colorectal Tissue (24) | Colorectal Carcinoma (36) | 2.300 | 8.956 | 6.82E-12 |
|  | Kaiser Colon | Colon (5) | Rectal Mucinous Adenocarcinoma (4) | 2.073 | 6.287 | 2.86E-4 |
|  | Hong Colorectal | Colon (12) | Colorectal Carcinoma (70) | 2.688 | 9.292 | 5.70E-9 |
|  | Skrzypczak Colorectal 2 | Colon (10) | Colon Carcinoma (5) | 2.397 | 6.912 | 1.17E-5 |
