# Supplementary material for: The expression profile and prognostic significance of eukaryotic translation elongation factors in different cancers
Source: PLoS One. 2018 Jan 17;13(1):e0191377. doi: 10.1371/journal.pone.0191377 (PMC5771626; doi:10.1371/journal.pone.0191377)
Supplement: S13 Table — (DOCX) [file pone.0191377.s021.docx]

**Supplementary Table 13: Analyses of elongation factors in lymphoma.**

| **Gene** | **Dataset** | **Normal (Cases)** | **Tumor (Cases)** | **Fold change** | **t-Test** | **p-value** |
| --- | --- | --- | --- | --- | --- | --- |
| EEF1A1 | Basso Lymphoma | B-Lymphocyte (5)/ Centroblast (5)/ Memory B-Lymphocyte (5)/ Naive Pregerminal Center B-Lymphocyte (5)/ Small Cleaved Follicle Center Cell (5) | Follicular Lymphoma (6) | 2.187 | 3.478 | 0.002 |
|  | Piccaluga Lymphoma | CD4-Positive T-Lymphocyte (5)/ CD8-Positive T-Lymphocyte (5)/ T-Lymphocyte (10) | Angioimmunoblastic T-Cell Lymphoma (6) | -3.494 | -9.512 | 4.11E-12 |
|  | Basso Lymphoma | B-Lymphocyte (5)/ Centroblast (5)/ Memory B-Lymphocyte (5)/ Naive Pregerminal Center B-Lymphocyte (5)/ Small Cleaved Follicle Center Cell (5) | Centroblastic Lymphoma (28) | -81.403 | -7.222 | 1.31E-8 |
|  | Storz Lymphoma | Skin (3)/Tonsil (3) | Marginal Zone B-Cell Lymphoma (5) | -2.735 | -3.883 | 0.003 |
| EEF1B2 | Brune Lymphoma | Centroblast (5)/Memory B-Lymphocyte (5)/Naive Pregerminal Center B-Lymphocyte (5)/ Plasma Cell (5)/ Small Cleaved Follicle Center Cell (5) | Follicular Lymphoma (5) | 2.489 | 8.928 | 4.01E-8 |
|  |  | Centroblast (5)/Memory B-Lymphocyte (5)/Naive Pregerminal Center B-Lymphocyte (5)/ Plasma Cell (5)/ Small Cleaved Follicle Center Cell (5) | Diffuse Large B-Cell Lymphoma (11) | 2.508 | 3.745 | 1.52E-4 |
|  |  | Centroblast (5)/Memory B-Lymphocyte (5)/Naive Pregerminal Center B-Lymphocyte (5)/ Plasma Cell (5)/ Small Cleaved Follicle Center Cell (5) | Burkitt's Lymphoma (5) | 3.217 | 7.779 | 4.48E-5 |
| EEF1G | Brune Lymphoma | Centroblast (5)/Memory B-Lymphocyte (5)/Naive Pregerminal Center B-Lymphocyte (5)/ Plasma Cell (5)/ Small Cleaved Follicle Center Cell (5) | Burkitt's Lymphoma (5) | 2.386 | 7.144 | 1.28E-5 |
|  |  | Centroblast (5)/Memory B-Lymphocyte (5)/Naive Pregerminal Center B-Lymphocyte (5)/ Plasma Cell (5)/ Small Cleaved Follicle Center Cell (5) | Diffuse Large B-Cell Lymphoma (11) | 2.092 | 5.629 | 6.95E-6 |
| EEF1D | Eckerle Lymphoma | Natural Killer Cell (5)/T-Lymphocyte (31)/T-Lymphocyte and Natural Killer Cell (5) | Anaplastic Large Cell Lymphoma, ALK-Negative (4) | 4.490 | 14.712 | 8.06E-13 |
|  |  | Natural Killer Cell (5)/T-Lymphocyte (31)/T-Lymphocyte and Natural Killer Cell (5) | Anaplastic Large Cell Lymphoma, ALK-Positive (5) | 2.101 | 6.074 | 5.92E-4 |
|  |  | Natural Killer Cell (5)/T-Lymphocyte (31)/T-Lymphocyte and Natural Killer Cell (5) | Classical Hodgkin's Lymphoma (4) | 2.661 | 4.543 | 0.005 |
|  | Choi Leukemia | CD4-Positive T-Lymphocyte | Acute Adult T-Cell Leukemia/Lymphoma (22) | 2.407 | 5.857 | 4.30E-6 |
|  | Brune Lymphoma | Centroblast (5)/ Memory B-Lymphocyte (5)/Naive Pregerminal Center B-Lymphocyte (5)/ Plasma Cell (5)/Small Cleaved Follicle Center Cell (5)/ Burkitt's Lymphoma (5) | Burkitt's Lymphoma (5) | 2.045 | 7.998 | 1.85E-6 |
|  |  | Centroblast (5)/ Memory B-Lymphocyte (5)/Naive Pregerminal Center B-Lymphocyte (5)/ Plasma Cell (5)/Small Cleaved Follicle Center Cell (5)/ Burkitt's Lymphoma (5) | Hodgkin's Lymphoma (12) | 2.027 | 5.211 | 1.48E-5 |
|  |  | Centroblast (5)/ Memory B-Lymphocyte (5)/Naive Pregerminal Center B-Lymphocyte (5)/ Plasma Cell (5)/Small Cleaved Follicle Center Cell (5)/ Burkitt's Lymphoma (5) | Follicular Lymphoma (5) | 3.141 | 6.298 | 3.66E-4 |
|  | Compagno Lymphoma | Germinal Center B-Lymphocyte (10)/Memory B-Lymphocyte (5)/Naive Pregerminal Center B-Lymphocyte (5) | Activated B-Cell-Like Diffuse Large B-Cell Lymphoma (17) | 2.118 | 7.012 | 4.53E-8 |
|  |  | Germinal Center B-Lymphocyte (10)/Memory B-Lymphocyte (5)/Naive Pregerminal Center B-Lymphocyte (5) | Diffuse Large B-Cell Lymphoma (44) | 2.157 | 8.271 | 1.20E-11 |
|  |  | Germinal Center B-Lymphocyte (10)/Memory B-Lymphocyte (5)/Naive Pregerminal Center B-Lymphocyte (5) | Germinal Center B-Cell-Like Diffuse Large B-Cell Lymphoma (9) | 2.330 | 4.735 | 3.74E-4 |
| EEF1E1 | Basso Lymphoma | B-Lymphocyte (5)/ Centroblast (5)/Memory B-Lymphocyte (5)/Naive Pregerminal Center B-Lymphocyte (5)/Small Cleaved Follicle Center Cell (5) | Burkitt's Lymphoma (17) | 3.807 | 6.676 | 3.62E-8 |
|  | Brune Lymphoma | Centroblast (5)/ Memory B-Lymphocyte (5)/Naive Pregerminal Center B-Lymphocyte (5)/Plasma Cell (5)/Small Cleaved Follicle Center Cell (5) | Diffuse Large B-Cell Lymphoma (11) | 2.036 | 4.850 | 1.31E-4 |
|  | Storz Lymphoma | Skin (3)/Tonsil (3) | Marginal Zone B-Cell Lymphoma (5) | -2.231 | -3.207 | 0.006 |
| EEF2 | Eckerle Lymphoma | Natural Killer Cell (5)/T-Lymphocyte (31)/T-Lymphocyte and Natural Killer Cell (5) | Anaplastic Large Cell Lymphoma, ALK-Positive (5) | 2.003 | 8.989 | 1.21E-5 |
|  |  | B-Lymphocyte (4)/CD4-Positive T-Lymphocyte (5)/ Germinal Center B-Lymphocyte (1)/Memory B-Lymphocyte (1)/Umbilical Cord Blood B-Lymphocyte (1)/ Umbilical Cord Blood T-Lymphocyte (1) | Follicular Lymphoma (7) | -2.774 | -5.319 | 2.43E-5 |
|  |  | B-Lymphocyte (4)/CD4-Positive T-Lymphocyte (5)/ Germinal Center B-Lymphocyte (1)/Memory B-Lymphocyte (1)/Umbilical Cord Blood B-Lymphocyte (1)/ Umbilical Cord Blood T-Lymphocyte (1) | T-Cell/Histiocyte-Rich Large B-Cell Lymphoma (4) | -2.129 | -4.667 | 0.003 |
