# Supplementary material for: The expression profile and prognostic significance of eukaryotic translation elongation factors in different cancers
Source: PLoS One. 2018 Jan 17;13(1):e0191377. doi: 10.1371/journal.pone.0191377 (PMC5771626; doi:10.1371/journal.pone.0191377)
Supplement: S14 Table — (DOCX) [file pone.0191377.s022.docx]

**Supplementary Table 14: Differential expression analyses of elongation factors in other cancers**

| **Gene** | **Dataset** | **Normal (Cases)** | **Tumor (Cases)** | **Fold change** | **t-Test** | **p-value** |
| --- | --- | --- | --- | --- | --- | --- |
| EEF1A1 | Welsh ovarian | Ovary (4) | Ovarian Serous Surface Papillary Carcinoma (28) | -2.114 | -6.020 | 7.27E-7 |
| EEF1A2 | Hendrix Ovarian | Ovary (4) | Ovarian Clear Cell Adenocarcinoma (8) | 2.538 | 6.319 | 7.69E-5 |
| EEF1E1 | Yoshihara Ovarian | Peritoneum (10) | Ovarian Serous Adenocarcinoma (43) | 2.146 | 6.007 | 6.87E-7 |
| EEF1A1 | Pei Pancreas | Pancreas (16) | Pancreatic Carcinoma (36) | -2.112 | -4.196 | 1.51E-4 |
| EEF1A2 | Logsdon Pancreas | Pancreas (5) | Pancreatitis (5) | -2.334 | -4.218 | 0.002 |
| EEF1B2 | Logsdon Pancreas | Pancreas (5) | Pancreatic Adenocarcinoma (10) | -2.270 | -11.654 | 2.41E-8 |
|  | Segara Pancreas | Pancreas (6) | Pancreatic Carcinoma (11) | -2.336 | -3.687 | 0.001 |
|  | Buchholz Pancreas | Pancreatic Duct (6) | Pancreatic Ductal Adenocarcinoma (8) | -3.245 | -2.976 | 0.008 |
| EEF1D | Segara Pancreas | Pancreas (6) | Pancreatic Carcinoma (11) | -4.797 | -4.541 | 4.27E-4 |
| EEF1E1 | Grutzmann Pancreas | Pancreatic Duct (11) | Pancreatic Ductal Adenocarcinoma (11) | -3.124 | -3.030 | 0.003 |
| EEF1A2 | Cutcliffe Renal | Fetal Kidney (3) | Clear Cell Sarcoma of the Kidney (14) | 3.828 | 9.603 | 1.06E-4 |
| EEF1B2 | Yusenko Renal | Fetal Kidney (2)/ Kidney (3) | Renal Wilms Tumor (4) | 2.414 | 5.166 | 7.71E-4 |
|  |  | Kidney (23) | Renal Pelvis Urothelial Carcinoma (8) | 2.649 | 10.345 | 1.17E-7 |
| EEF1D | Cutcliffe Renal | Fetal Kidney (3) | Renal Wilms Tumor (18) | 2.185 | 4.181 | 0.003 |
|  | Cutcliffe Renal | Fetal Kidney (3) | Clear Cell Sarcoma of the Kidney (14) | 7.274 | 11.335 | 0.002 |
|  | Gumz Renal | Kidney (10) | Clear Cell Renal Cell Carcinoma (10) | -2.215 | -5.378 | 2.49E-5 |
| EEF1E1 | Yusenko Renal | Fetal Kidney (2)/ Kidney (3) | Chromophobe Renal Cell Carcinoma (4) | -2.783 | -5.018 | 0.006 |
| EEF1A2 | Estilo Head-Neck | Tongue (26) | Tongue Squamous Cell Carcinoma (31) | -7.801 | -4.831 | 1.11E-5 |
|  | FriersonHF Salivary-gland | Salivary Gland (6) | Salivary Gland Adenoid Cystic Carcinoma (16) | -3.468 | -3.689 | 8.58E-4 |
| EEF1B2 | Schlingemann Head-Neck | Hypopharynx (3)/ Oropharynx (1) | Hypopharyngeal Squamous Cell Carcinoma (4) | -2.008 | -4.725 | 0.002 |
| EEF1D | Pyeon Multi-cancer | Cervix Uteri (8)/ Oral Cavity (9)/Palate (1) / Tonsil (4) | Floor of the Mouth Carcinoma (5) | 3.751 | 4.859 | 2.82E-5 |
|  |  | Cervix Uteri (8)/ Oral Cavity (9)/Palate (1) / Tonsil (4) | Tongue Carcinoma (15) | 3.031 | 4.139 | 1.78E-4 |
| EEF1E1 | Roessler Liver 2 | Liver (220) | Hepatocellular Carcinoma (225) | 2.377 | 21.280 | 6.11E-67 |
|  | Roessler Liver | Liver (21) | Hepatocellular Carcinoma (22) | 2.235 | 4.913 | 8.11E-6 |
