# Supplementary material for: The expression profile and prognostic significance of eukaryotic translation elongation factors in different cancers
Source: PLoS One. 2018 Jan 17;13(1):e0191377. doi: 10.1371/journal.pone.0191377 (PMC5771626; doi:10.1371/journal.pone.0191377)
Supplement: S15 Table — Abbreviations: OS: overall survival; RFS: relapse free survival; DMFS: distant metastasis free survival; PPS: post progression survival; HR: Hazard radio; CI: Confidence interval. p-values ≤ 0.05 were considered statistically significant and have been denoted in bold. (DOCX) [file pone.0191377.s023.docx]

**Supplementary Table 15: Kaplan-Meier plotter data showing the correlation between different elongation factors and survival outcomes in ovarian cancer**

| **Gene** | **Dataset/**  **Affymetrix ID** | **Survival outcome** | **No. of Cases** | **HR** | **95% CI** | **p-value** |
| --- | --- | --- | --- | --- | --- | --- |
| EEF1A1 | 227708_at | OS | 1657 | 0.99 | 0.81-1.21 | 0.91 |
|  |  | PFS | 1436 | 0.88 | 0.73-1.07 | 0.2 |
|  |  | PPS | 782 | 1.05 | 0.83-1.33 | 0.69 |
| EEF1A2 | 204540_at | OS | 1657 | 0.96 | 0.85-1.1 | 0.56 |
|  |  | RFS | 1436 | 0.89 | 0.79-1.01 | 0.083 |
|  |  | PPS | 782 | 0.99 | 0.83-1.17 | 0.89 |
| EEF1B2 | 200705_s_at | OS | 1657 | 0.97 | 0.85-1.1 | 0.63 |
|  |  | PFS | 1436 | 0.93 | 0.82-1.05 | 0.23 |
|  |  | PPS | 782 | 0.87 | 0.74-1.03 | 0.12 |
| EEF1G | 211345_x_at | OS | 1657 | 0.87 | 0.76-0.98 | **0.027** |
|  |  | PFS | 1436 | 0.79 | 0.7-0.9 | **0.00027** |
|  |  | PPS | 782 | 0.92 | 0.78-1.09 | 0.35 |
| EEF1D | 203113_s_at | OS | 1657 | 0.91 | 0.8-1.04 | 0.17 |
|  |  | RFS | 1436 | 0.93 | 0.92-1.06 | 0.29 |
|  |  | PPS | 782 | 0.93 | 0.79-1.1 | 0.41 |
| EEF1E1 | 204905_s_at | OS | 1657 | 0.96 | 0.85-1.1 | 0.57 |
|  |  | PFS | 1436 | 1.06 | 0.94-1.21 | 0.33 |
|  |  | PPS | 782 | 0.97 | 0.82-1.14 | 0.7 |
| EEF2 | 200094_s_at | OS | 1657 | 0.89 | 0.78-1.01 | 0.066 |
|  |  | RFS | 1436 | 0.95 | 0.84-1.08 | 0.46 |
|  |  | PPS | 782 | 0.97 | 0.82-1.14 | 0.7 |
